# Cardiolipin Biosynthesis Genes Are Not Required for *Salmonella enterica* Serovar Typhimurium Pathogenesis in C57BL/6J Mice

**DOI:** 10.1128/spectrum.02617-21

**Published:** 2022-05-31

**Authors:** Melina B. Cian, Joshua A. Mettlach, Aaron E. Zahn, Nicole P. Giordano, Keaton E. Minor, Michael McClelland, Zachary D. Dalebroux

**Affiliations:** a Department of Microbiology and Immunology, University of Oklahoma Health Sciences Center, Oklahoma City, Oklahoma, USA; b Department of Microbiology and Molecular Genetics, University of California, Irvine, Irvine, California, USA; Emory University School of Medicine

**Keywords:** cardiolipin, ClsA, ClsB, ClsC, phospholipase-D, macrophage, inflammasome, interleukin-1 beta, pyroptosis, tumor necrosis factor alpha, Toll-like receptor four, gastroenteritis, bacteremia, interleukins, lipopolysaccharide, phospholipids

## Abstract

Salmonella enterica serovar Typhimurium is an intracellular pathogen that parasitizes macrophages from within a vacuole. The vacuolar environment prompts the bacterium to regulate the lipid composition of the outer membrane (OM), and this influences host inflammation. *S*. Typhimurium regulates the levels of acidic glycerophospholipids known as cardiolipins (CL) within the OM, and mitochondrial CL molecules can prime and activate host inflammasomes. However, the contribution of *S*. Typhimurium’s CL biosynthesis genes to intracellular survival, inflammasome activation, and pathogenesis had not been examined. *S*. Typhimurium genes encode three CL synthases. Single, double, and triple mutants were constructed. Similar to other *Enterobacteriaceae*, ClsA is the primary CL synthase for *S*. Typhimurium during logarithmic growth, while ClsB and ClsC contribute CL production in stationary phase. It was necessary to delete all three genes to diminish the CL content of the envelope. Despite being devoid of CL molecules, Δ*clsABC* mutants were highly virulent during oral and systemic infection for C57BL/6J mice. In macrophages, Δ*clsA*, Δ*clsB*, Δ*clsC*, and Δ*clsAC* mutants behaved like the wild type, whereas Δ*clsAB*, Δ*clsBC*, and Δ*clsABC* mutants were attenuated and elicited reduced amounts of secreted interleukin-1 beta (IL-1β), IL-18, and lactate dehydrogenase. Hence, when *clsA* and *clsC* are deleted, *clsB* is necessary and sufficient to promote intracellular survival and inflammasome activation. Similarly, when *clsB* is deleted, *clsA* and *clsC* are necessary and sufficient. Therefore, the three CL synthase genes cooperatively and redundantly influence *S*. Typhimurium inflammasome activation and intracellular survival in C57BL/6J mouse macrophages but are dispensable for virulence in mice.

**IMPORTANCE** Salmonella enterica serovar Typhimurium is a pathogenic Gram-negative bacterium that regulates the cardiolipin (CL) and lipopolysaccharide (LPS) composition of the outer membrane (OM) during infection. Mitochondrial CL molecules activate the inflammasome and its effector caspase-1, which initiates an inflammatory process called pyroptosis. Purified bacterial CL molecules also influence LPS activation of Toll-like receptor 4 (Tlr4). *S*. Typhimurium resides within macrophage vacuoles and activates Tlr4 and the inflammasome during infection. However, the contribution of the three bacterial CL synthase genes (*cls*) to microbial pathogenesis and inflammation had not been tested. This study supports that the genes encoding the CL synthases work coordinately to promote intracellular survival in macrophages and to activate the inflammasome but do not influence inflammatory cytokine production downstream of Tlr4 or virulence in C57BL/6J mice. The macrophage phenotypes are not directly attributable to CL production but are caused by deleting specific combinations of *cls* gene products.

## INTRODUCTION

*Enterobacteriaceae* is a large family of pathogenic and nonpathogenic Gram-negative bacteria that typically reside within the gastrointestinal tract of mammals. The cell envelope of these microbes consists of inner (IM) and outer membranes (OM) that are partitioned by a periplasmic space and thin peptidoglycan cell wall (1). The integrated architecture of the individual layers for the envelope is critical for bacterial physiology and pathogenesis. Glycerophospholipids (GPLs) are among the essential molecular building blocks for the envelope that comprise the inner and outer leaflets of the IM and the inner leaflet of the OM (2). The OM is an asymmetric lipid bilayer that contains an outer leaflet, which consists primarily of lipopolysaccharides (LPS) (3). Bilayer asymmetry and the biochemistry of LPS molecules confer barrier properties to the OM that prevent the passage of noxious molecules and protect the bacterium against hazards in the environment (4). Salmonella enterica serovar Typhimurium is a facultative intracellular pathogen that regulates the GPL and LPS composition of the OM to resist cell-autonomous host defense and to establish a vacuolar survival niche in macrophages (5–7).

GPLs are amphipathic molecules that are necessary for bacteria to maintain OM-lipid asymmetry, promote OM-barrier function, and enhance antibiotic resistance (8). GPLs form specific interactions with cell envelope proteins and modulate protein function (9, 10). The predominant GPLs for *Enterobacteriaceae* are the phosphatidylethanolamines (PE; ~70 to 80% of the total GPL content), phosphatidylglycerols (PGl; ~10 to 20%), di-phosphatidylglycerols or cardiolipins (CL; ~2 to 10%), acyl-phosphatidylglycerols (acyl-PGl; <1%), and phosphatidic acids (PA; <1%) (11). The relative abundance of these molecules varies as a function of media composition, temperature, osmolarity, pH, and bacterial growth phase (12–14). Enzymes that synthesize PE and PGl molecules are highly conserved and essential for bacterial viability, while enzymes that synthesize CL and acyl-PGl are redundant and dispensable (15).

**FIG 1:**
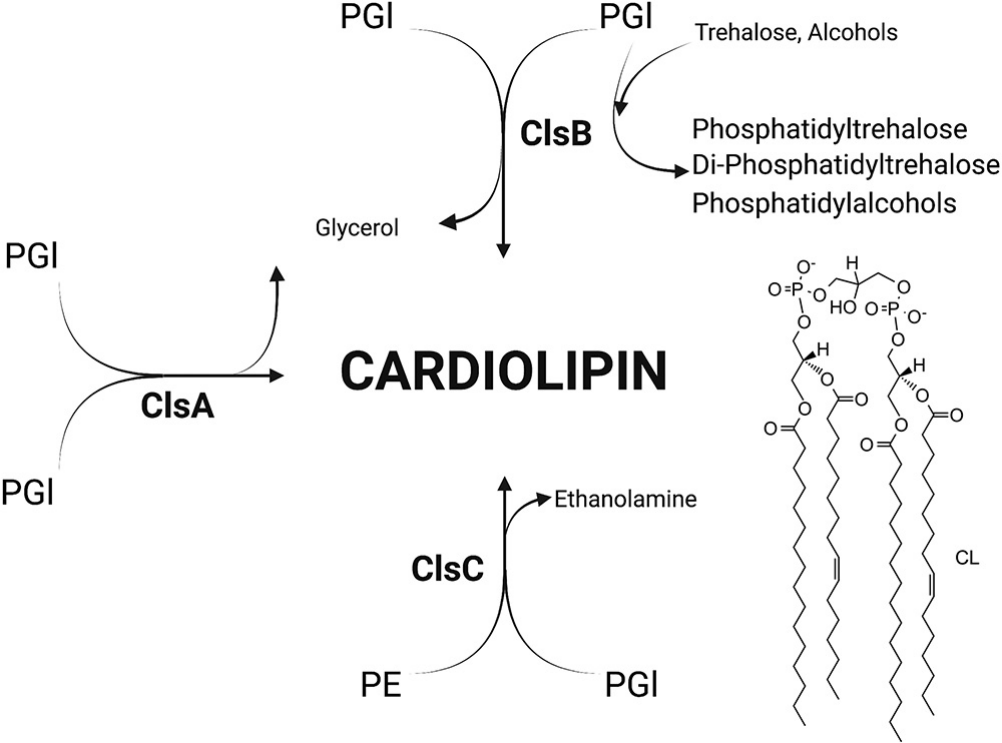
Salmonella enterica serovar Typhimurium (*S*. Typhimurium) encodes three cardiolipin (CL) synthases that contribute to CL biosynthesis. The schematic highlights the convergence of the three pathways for cardiolipin (CL) biosynthesis in enterobacterial microorganisms. ClsA catalyzes the transfer of a phosphatidyl group from one phosphatidylglycerol (PGl) molecule to a second PGl molecule to generate diphosphatidylglycerol, also known as CL, and glycerol. ClsB produces CL from two PGl donor molecules through a similar transphosphatidylation reaction. Unlike ClsA and ClsC, ClsB has broad substrate specificity and also catalyzes synthesis of phosphatidyltrehalose, diphosphatidyltrehalose, phosphatidylalcohols, and PGl though transphosphatidylation. ClsC catalyzes a transphosphatidylation reaction between one phosphatidylethanolamine (PE) and one PGl molecule, which generates CL and ethanolamine. (This figure was created with BioRender.com.)

Unlike other families of bacteria, *Enterobacteriaceae* carry three CL synthases, ClsA, ClsB, and ClsC, which possess characteristic phospholipase-D motifs (Fig. 1) (16–18). In addition to functional redundancy, the three CL synthases likely allow biochemical features not found in other families of bacteria. ClsA is the predominant synthase during logarithmic growth that uses two PGl precursor substrates to produce one CL molecule (19, 20). ClsC generates CL from single PGl and single PE precursor substrates and contributes to the CL pool during stress (e.g., in stationary phase or in high osmolarity medium) (16). ClsB synthesizes a single CL molecule from two PGl molecules and, like ClsC, is also activated during stationary-phase stress (17). ClsB is a promiscuous enzyme that can also synthesize PGl, phosphatidylalcohols, phosphatidyltrehalose (PT), and diphosphatidyltrehalose (diPT) (Fig. 1) (18, 21). PT and diPT are immunologically important glycophospholipids produced by ClsB in S. enterica serovars, which contain a polar head-group structure similar to that of the trehalose dimycolates generated by *Mycobacteria* (22). Therefore, ClsA and ClsC are largely dedicated to CL biosynthesis, while ClsB likely has additional biochemical roles.

Purified bacterial CL molecules interact with host-pattern recognition receptors, such as the membrane-bound Toll-like receptor 4 (Tlr4) complex (23). Tlr4 engages the lipid A moiety of bacterial LPS molecules and activates nuclear factor kappa beta (NF-κβ) to induce transcription of inflammatory cytokines like tumor necrosis factor alpha (TNF-α), prointerleukin-1 beta (pro-IL-1β), and prointerlukin-18 (pro-IL-18) (24, 25). Tlr4 is highly activated by hexacylated lipid A molecules, and purified bacterial CL molecules with saturated fatty acids can decrease Tlr4 activation by lipid A molecules in macrophages (26, 27). Therefore, it has been suggested that bacterial CL molecules might influence the host-Tlr4 response to infection.

Inflammasomes are cytosolic multiprotein complexes that initiate innate immune signaling by recruitment of caspase-1. Mitochondrial CL molecules bind to the Nucleotide-binding and oligomerization domain (NOD-), Leucine-Rich repeats (LRR-) and pyrin domain-containing protein 3 (NLRP3) protein, which binds the apoptosis-associated speck-like protein (ASC) and forms a complex with caspase-1 as part of inflammasome priming and activation (28, 29). Caspase-1 itself also binds to mitochondrial CL molecules that have become externalized on the outer leaflet of the OM for the organelle (30). A variety of signals can activate NLRP3-Acs-caspase-1, including K^+^ ion efflux (31, 32). Inflammasome activation induces caspase-1 to cleave pro-IL-1B, pro-IL-18, and gasdermin D (33). The active form of gasdermin D is a pore-forming fragment whose membrane oligomerization results in the secretion of the mature forms of IL-1β and IL-18, which drives a potent proinflammatory lytic cell death, termed pyroptosis (34–36). Additionally, ninjurin-1 (NINJ-1) mediates plasma membrane rupture after the cascade of pyroptotic events and is necessary for the release of large damage-associated molecular patterns (DAMPs), such as high-mobility group box-1 (HMGB-1) and lactate dehydrogenase (LDH) (37). Oxidative metabolism of *S*. Typhimurium and production of bacterial citrate activate caspase-1 through NLRP3-Asc, but the mechanism is not defined (38). *S*. Typhimurium can also activate the NLRC4-NAIP5-caspase-1 inflammasome through the production of flagellin and a type-three secretion system rod protein, PrgJ (39). Furthermore, *S*. Typhimurium flagellin can activate both NLRC4-NAIP5 and NLRP3-Asc inflammasomes in a human cell model (40). Although their role has been predicted, the contribution of *S*. Typhimurium CL molecules to inflammasome activation has not been examined.

Unlike mitochondria, *S*. Typhimurium is typically bound by a vacuolar membrane in macrophages. The harsh conditions of the vacuole elicit *S*. Typhimurium to regulate the CL content of the OM, but the role of the CL biosynthesis genes had not been examined (5, 14, 41, 42). We provide data to support the observation that S. Typhimurium carries three genes that encode three CL synthases. which are each independently capable of CL production. Deleting all three enzymes eliminates the CL content of the envelope but has minimal impact on the virulence of *S*. Typhimurium in C57BL/6J mice following oral or systemic infection. During intracellular survival in macrophages, Δ*clsA*, Δ*clsB*, Δ*clsC*, and Δ*clsAC* mutants behave like the wild type, whereas Δ*clsAB*, Δ*clsBC*, and Δ*clsABC* mutants are attenuated and elicit reduced amounts of secreted IL-1β, IL-18, and LDH. The data indicate that when *clsA* and *clsC* are deleted, *clsB* is necessary and sufficient to promote intracellular survival and inflammasome activation. Similarly, when *clsB* is deleted, *clsA* and *clsC* are necessary and sufficient. Adding back *clsA* or *clsB* to the triple mutant bacteria restores CL production but does not rescue the macrophage phenotypes. Therefore, the observed phenotypes are not caused by alterations in CL content.

## RESULTS

### *clsA*, *clsB*, and *clsC* contribute to CL biosynthesis in *S*. Typhimurium.

**FIG 2:**
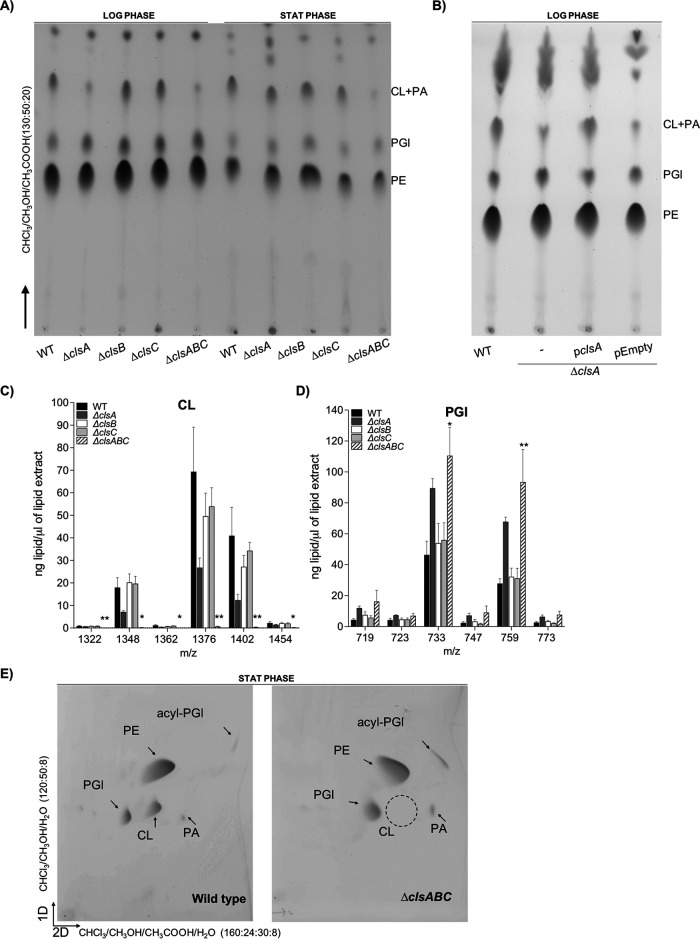
*S*. Typhimurium requires *clsA* for CL biosynthesis during logarithmic growth, while *clsB* and *clsC* contribute to CL production during stationary phase. (A) The wild-type (WT) and *cls*-mutant *S*. Typhimurium isolates were cultured to either the logarithmic (LOG) or stationary (STAT) phase of growth. Glycerophospholipids (GPL) were extracted from the total membrane fractions and separated by one-dimensional thin-layer chromatography (1D-TLC). PA, phosphatidic acid. (B) The GPLs derived from total membrane of fractions of log-phase WT, Δ*clsA*, and Δ*clsA* bacteria carrying either an empty vector or a vector encoding the *clsA* operon were separated and visualized by 1D-TLC. (C and D) GPLs were extracted from the total membranes of the WT and *cls*-mutant *S*. Typhimurium at the stationary phase of growth and quantified by normal-phase liquid-chromatography tandem mass spectrometry (LC-MS/MS). The quantities from four independent biological replicates are presented as the average amount of lipid per extract ± standard error of the mean (SEM). A one-way analysis of variance (ANOVA) followed by Bonferroni posttest was used. Asterisks indicate a significant difference for the Δ*clsABC* triple mutant relative to the wild type (*, *P* < 0.05; **, *P* < 0.01). (E) Two-dimensional (2D)-TLC analysis of the GPLs that were extracted from the total membranes of the wild-type and Δ*clsABC*-mutant *S*. Typhimurium. The empty circle denotes the predicted migration position of CL in the Δ*clsABC*-mutant extract; however, this genotype is devoid of detectable CL. acyl-PGl, acylphosphatidylgycerol.

To study the contribution of *S*. Typhimurium’s three CL synthases to CL biosynthesis and bacterial virulence, we generated single, double, and triple mutant bacteria (Table 1). Bacteria were cultured to either the logarithmic (log) or stationary phase (stat) of growth and lysed. Membranes were collected, and the GPL molecules were extracted. Differences in GPL abundance were assessed by thin-layer chromatography (TLC) and liquid-chromatography tandem mass spectrometry (LC-MS/MS) (Fig. 2). To identify each lipid family on a TLC plate, commercial standards of PE, PGl, CL, and PA were chromatographed, and their retention factors were compared against those of the lipids that were present within the total membrane samples from the bacteria (see Fig. S1A in the supplemental material). In the log phase of growth, the membranes of the Δ*clsA* mutant contained diminished amounts of CL compared to the wild type, while the Δ*clsB* and Δ*clsC* mutants produced amounts of CL equivalent to those of the wild type by TLC analysis (Fig. 2A). Supplying the *clsA* operon on a plasmid restored CL production to the Δ*clsA* mutant in the log phase of growth (Fig. 2B). In the stationary phase of growth, the CL levels for the wild type and the Δ*clsA*, Δ*clsB*, and Δ*clsC* mutants were qualitatively similar by TLC analysis (Fig. 2A). These results were corroborated by LC-MS/MS analysis. The Δ*clsA* mutant routinely measured a reduction in CL molecules in stationary phase by LC-MS/MS, but the differences were not statistically significant from the wild-type strain (Fig. 2C). By TLC, the Δ*clsAB*, Δ*clsBC*, and Δ*clsAC* double mutants also showed a reduction in the levels of CL compared to those of the wild type and the single-mutant genotypes; nevertheless, quantitative examination by LC-MS/MS revealed that the differences were not statistically significant from the wild type (Fig. S1B and C).

**TABLE 1:** Bacterial strains and plasmids

Strain or plasmid	Wild type or mutant	Genotype and/or note(s)	Reference
Salmonella enterica serovar Typhimurium 14028S			
ZD004	Wild-type *wza-lacZ*	Wild-type *wza-lacZ-cat*	74
	Wild type	Wild type	ATCC
ZD0027	Δ*clsA*	*clsA::tetRA*	This study
ZD0028	Δ*clsB*	*clsB::kan*	This study
ZD0029	Δ*clsB*	*clsB::cat*	This study
ZD0030	Δ*clsC*	*clsC::kan*	This study
ZD0031	Δ*clsC*	*clsC::cat*	This study
ZD0032	Δ*clsAB*	*clsA::tetRA clsB::kan*	This study
ZD0033	Δ*clsAC*	*clsA::tetRA clsC::cat*	This study
ZD0034	Δ*clsBC*	*clsB::cat clsC::kan*	This study
ZD0035	Δ*clsABC*	*clsA::tetRA clsB::cat clsC::kan*	This study
Escherichia coli			
DH5α		Transformation and cloning intermediate	ATCC
Plasmids			
pKD46		This plasmid encodes the lambda phage-red system and the *bet*, *exo*, and *gam* genes under control of the arabinose inducible PBAD promoter.	71
pWSK29		Low copy no. cloning vector; Amp^R^	75
pWSK29-*clsA-yciU*		Encodes whole *clsA* operon; Amp^R^	This study
pWSK29-*ybhP-clsB-ybhN*		Encodes whole *clsB* operon; Amp^R^	This study
pWSK29-*ybhP-clsB^H290A^-ybhN*		Encodes whole *clsB* operon with a substitution Ala for a His at residue 290, creating a catalytically inactive ClsB enzyme; Amp^R^	This study

Codeletion of all three CL synthase genes resulted in a severe decrease in CL levels relative to those of the wild type in both growth phases (Fig. 2A). However, lipid material with a retention factor similar to that of CL and PA was still observed in the membrane extracts from the Δ*clsABC* mutant by TLC (Fig. 2A, Fig. S1A). To further separate the CL and PA molecules and determine if this material could still be CL, we performed 2D-TLC. We observed that the lipid material that comigrated with CL by 1D-TLC was likely PA, since by 2D-TLC the Δ*clsABC* mutant extracts were devoid of lipids with a retention factor similar to that of CL yet contained lipid material with a retention factor similar to that of PA relative to the wild-type extracts, which contained both CL and PA (Fig. 2E). Furthermore, CL molecules were near the detection limit for the triple mutants by LC-MS/MS analysis and the levels were significantly reduced relative to those of the wild-type and single-mutant genotypes (Fig. 2D). The Δ*clsABC* mutant also measured a significant increase in two PGl molecules, *m/z* 733 and 759, relative to the wild-type strain in stationary phase (Fig. 2B). Therefore, *clsA* is the primary CL synthase for *S*. Typhimurium in the log phase of growth, while *clsB* and *clsC* contribute to CL biosynthesis in stationary phase, and removal of all three enzymes diminishes the CL content of the envelope regardless of the growth phase.

### *S*. Typhimurium does not require *clsABC* to infect mice systemically or to cause lethal bacteremia in C56Bl/6J animals.

**FIG 3:**
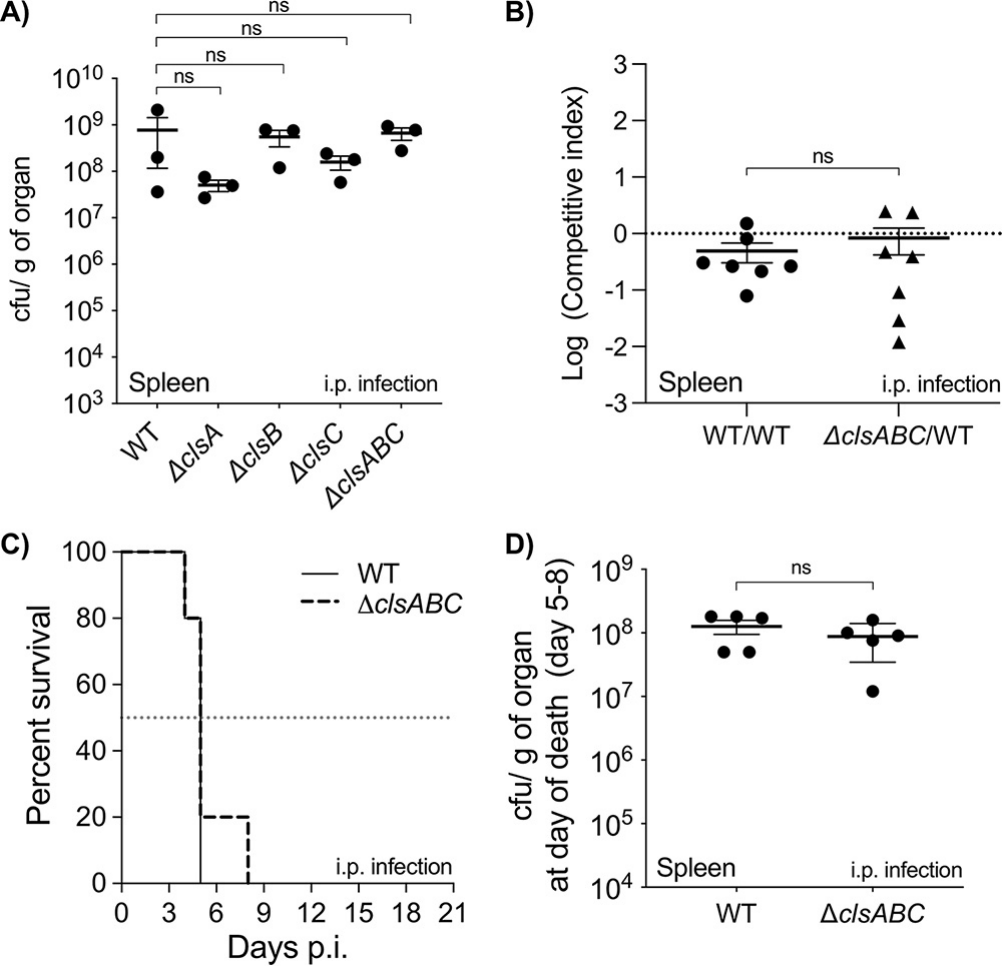
*clsABC-*dependent CL biosynthesis is not necessary for *S*. Typhimurium to infect mice following intraperitoneal injection and is dispensable for infection lethality. (A) C57BL/6J mice were intraperitoneally (i.p.) injected with 5 × 10^5^ CFU of the WT and *cls*-mutant *S*. Typhimurium. After 2 days, the mice were euthanized and CFU were enumerated from spleen homogenates. Data are shown as the mean number of organisms per weight of organ tissue ± SEM. Three mice were infected per genotype. A one-way ANOVA was executed to determine significance; however, the mutant CFU values were not statistically different (ns) from those of the wild type. (B) For the competition assay, two bacterial genotypes were mixed in a 1:1 ratio. The mixture, which contained roughly 5 × 10^5^ CFU, was used to infect C57BL/6J mice by i.p. injection. The control condition was a wild type versus wild type competition, which included a mixture of a wild-type strain that contained a chromosomally integrated *wza-lacZ-cat* reporter construct (chloramphenicol-resistant) and a wild-type strain that did not contain the reporter. The experimental condition consisted of a Δ*clsABC* versus wild type competition. Seven mice were infected for each condition. A one-way ANOVA followed by Dunnett’s posttest was used to test significance. A significant difference was not observed between the Δ*clsABC* versus wild type competition and the wild type versus wild type competition. (C) The survival of the C57BL/6J mice was monitored until the day of death, or for 21 days after i.p. infection, whichever was first. Mice were inoculated with roughly 5 × 10^3^ CFU of the wild-type strain (*n* = 5) or the Δ*clsABC* mutant (*n* = 5). Data are shown as percent survival. The median survival was 5 days for both strains. Statistical comparison of the curves was done using the log-rank (Mantel-Cox) test, and no statistical difference was found between two conditions. (D) At the day of death, the CFU/g of spleen was quantified for the mice that were infected with the wild type (*n* = 5 mice) or the Δ*clsABC* mutant (*n* = 5). Data are shown as the mean CFU/g ± SEM. A one-way ANOVA was executed to determine significance; however, the mutant CFU values were not statistically different from those of the wild type.

Data from our lab and others suggested that *S*. Typhimurium regulates the CL content of the OM to promote survival in host tissues; however, a bacterial genotype devoid of CL molecules had not been examined (5, 14, 41, 42). Therefore, we were keen to test whether *clsABC*-dependent CL production was necessary for bacterial pathogenesis in mice. Male and female C57BL/6J animals were intraperitoneally (i.p.) inoculated with approximately 5.0 × 10^5^ CFU of wild-type and Δ*clsA*, Δ*clsB*, Δ*clsC*, and Δ*clsABC* mutant *S*. Typhimurium. At 48 h, the mice were euthanized and the spleens and livers were homogenized. The organs from the mice infected with the wild type or single or triple mutant contained statistically identical numbers (1.0 × 10^7^ to 1.0 × 10^9^ CFU/gram of tissue) of surviving bacteria at 2 days postinfection, suggesting that the mutants were not attenuated (Fig. 3A, Fig. S2).

As a more sensitive measure of virulence attenuation, we performed a competition assay and coinoculated mice via the intraperitoneal route with both the wild type and the Δ*clsABC* mutant. At 2 days, the bacterial loads for the wild type versus Δ*clsABC* mutant competition were statistically invariant from wild type versus wild-type control competition, which suggested that the wild type and Δ*clsABC* mutants were equivalently fit for their ability to colonize mice systemically (Fig. 3B). Therefore, the *clsABC* genes are dispensable for *S*. Typhimurium’s ability to infect C57BL/6J mice under these conditions.

Finally, we tested whether the Δ*clsABC*-mutant infections were lethal to the animals. For this assay, male and female C57BL/6J mice were intraperitoneally infected with 5 × 10^3^ CFU and monitored for signs of morbidity until they reach the criteria for euthanasia, or up to 21 days postinfection, whichever occurred first. As for the colonization studies, the lethality assay showed that the wild type and Δ*clsABC* mutants killed mice at statistically identical rates, and the infections were lethal by between 5 and 8 days (Fig. 3C). Further, the spleens from the deceased animals contained statistically identical levels of wild-type and Δ*clsABC*-mutant salmonellae (Fig. 3D). Therefore, *S*. Typhimurium does not require *clsABC* to infect and kill C57BL/6J mice after intraperitoneal inoculation.

### *clsABC* are not necessary for gastrointestinal colonization of C56Bl/6J mice.

**FIG 4:**
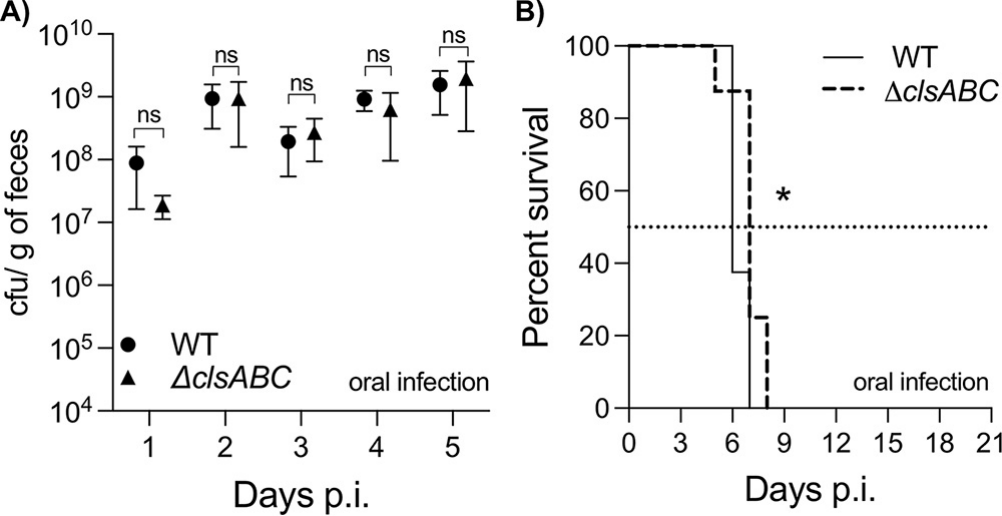
*clsABC*-dependent CL biosynthesis is dispensable for *S*. Typhimurium to colonize the gastrointestinal tract of mice. (A) Five C57BL/6J mice per group were infected by oral gavage with 1 × 10^8^ CFU of the wild type or the Δ*clsABC* mutant. Each point on the graph represents the mean CFU/g of stool ± SEM. A two-way ANOVA was done to assess significance, and no statistical difference (ns) was observed between the strains at each time point. (B) The survival of C57BL/6J-streptomycin-treated mice was monitored for 21 days after intragastric infection with roughly 1 × 10^5^ CFU of the wild-type strain (*n* = 8) and Δ*clsABC* (*n* = 8). Data are shown as percent survival. A Log-rank (Mantel-Cox) test was executed to determine significance, and a statistical difference, *P* < 0.05 (*), was found between curves. The median days of survival for the mice infected with the wild-type and Δ*clsABC*-mutant bacteria were 6 and 7 days, respectively.

The natural route of infection for *S*. Typhimurium is oral ingestion. Since we could not identify a role during systemic pathogenesis, we next sought to determine whether *clsABC* contributed to promoting gastrointestinal colonization. Briefly, streptomycin-treated C57BL/6J animals were intragastrically inoculated with 1.0 × 10^8^ CFU of the wild-type and triple mutant bacteria, and fecal samples were collected daily to determine colonization differences. Over the infection period, the mice inoculated with the wild-type and Δ*clsABC-*mutant genotypes shed statistically identical amounts of bacteria in their feces (Fig. 4A). Therefore, *clsABC* is not necessary for *S*. Typhimurium to colonize and survive within the gastrointestinal tract of these animals.

In the streptomycin-treated C57BL/6J mouse model of gastroenteritis, *S*. Typhimurium eventually disseminates into the lymphatic system, colonizes the systemic organs, and causes lethality (43–45). To assess whether *clsABC* contributes to the ability of *S*. Typhimurium to kill mice after oral infection, infected animals were monitored for signs of morbidity and mortality. Mice infected with the wild-type strain had a median survival of 6 days, while mice infected with the Δ*clsABC* mutant had a median survival of 7 days (Fig. 4B). Although modest, this difference was statistically significant and suggested a possible minor role for *clsABC* in *S*. Typhimurium-induced lethality following oral infection.

### The Δ*clsABC*, Δ*clsAB*, and Δ*clsBC* mutants are attenuated in C57BL/6J mouse macrophages, while the Δ*clsA*, Δ*clsB*, Δ*clsC*, and Δ*clsAC* mutants are not.

**FIG 5:**
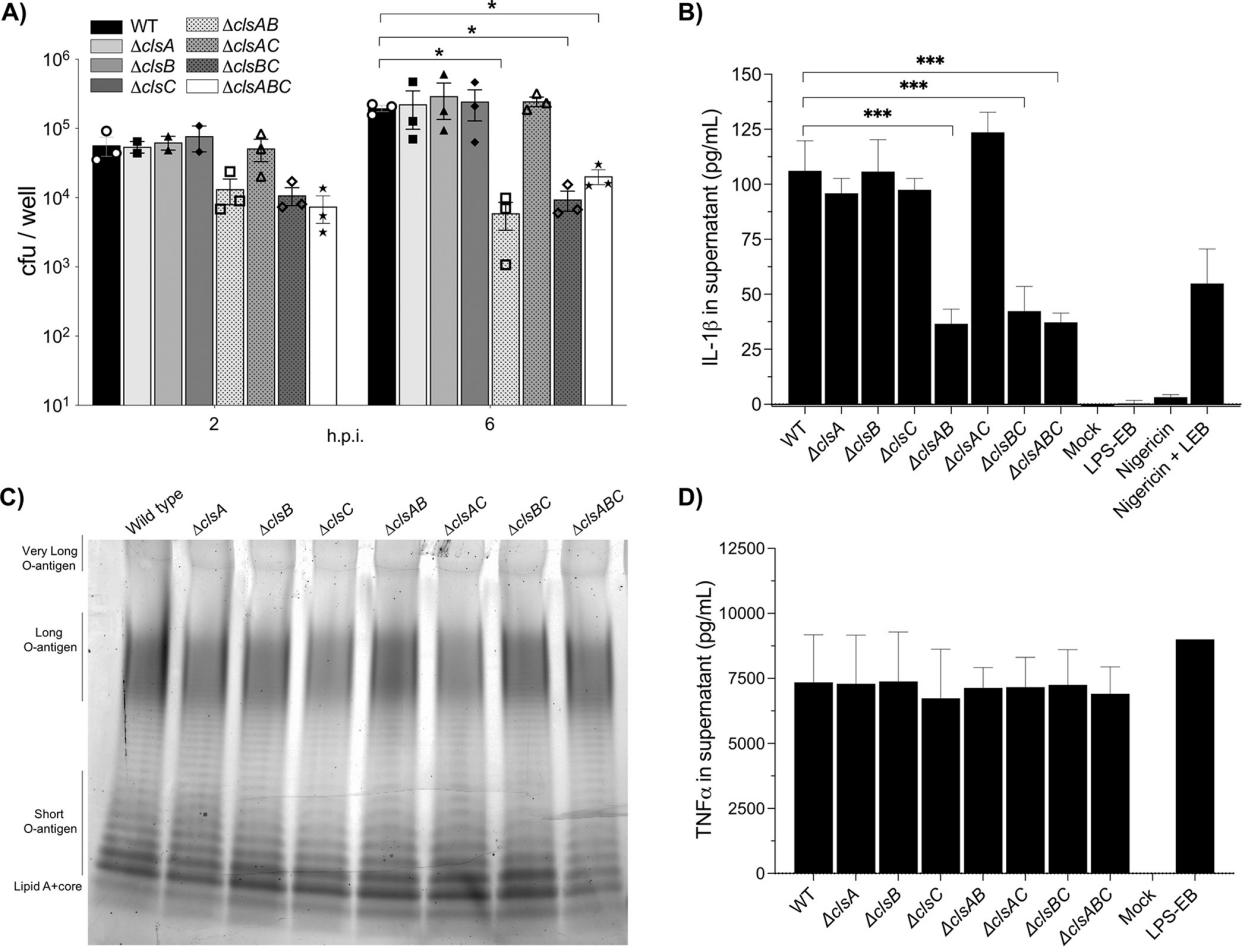
*clsB* is necessary to promote *S*. Typhimurium infection for primary bone marrow-derived mouse macrophages (BMDMs) and IL-1β secretion when *clsA* or *clsC* is deleted. (A) Macrophages were infected with the WT and *cls*-mutant bacteria. The surviving intracellular bacteria were enumerated at 2 and 6 h postinfection (hpi). Triplicate wells were infected for each genotype. The graph depicts the individual mean number of CFU per well ± SEM for three independent experiments, except for Δ*clsA*, Δ*clsB*, and Δ*clsC*, which were tested only twice at 2 hpi. A two-way ANOVA followed by Dunnett’s posttest was used to determine statistical significance. Significant differences relative to the wild type are indicated by *, *P* < 0.05. (B) The supernatants of the infected cells depicted in panel A were collected at 6 hpi, and the levels of secreted IL-1β were freshly quantified using a sandwich ELISA. As a positive control for inflammasome induction and IL-1β secretion, macrophages were treated with the potassium ionophore, nigericin, and lipopolysaccharide from Escherichia coli O111:B4 (LPS-EB). Three wells were infected per bacterial genotype, and the data are shown as the average pg/mL ± SD of secreted IL-1β. The graph represents one of three independent experiments. To determine statistical significance, a one-way ANOVA was executed followed by Dunnett’s posttest. Significant differences are indicated (***, *P* < 0.001). (C) LPS molecules from stationary-phase cultures of WT and *cls*-mutant only were extracted, electrophoresed, and stained with ProQ300 Emerald. The gel represents one of three independent experiments. (D) The supernatants of the infected cells depicted in panel A were collected at 6 hpi, and the levels of secreted TNF-α were freshly quantified using a sandwich ELISA. LPS-EB was added at a concentration of 10 ng/mL to the macrophages as a positive control. Three wells were infected per bacterial genotype, and the data are shown as the average pg/mL ± SD of secreted TNF-α. The graph represents one of three independent experiments. To determine statistical significance, a one-way ANOVA was executed followed by Dunnett’s posttest. No significant difference was observed between the wild-type and mutant genotypes.

*S*. Typhimurium survives in macrophages as part of causing disease in mammals (46, 47). Macrophages secrete inflammatory cytokines that influence the host response to infection. Therefore, we used primary bone marrow-derived macrophages to probe for differences in bacterial survival and macrophage cytokine production. Δ*clsABC* mutants were modestly attenuated in macrophages, and one-log reductions were measured repeatedly at 2 and 6 h postinfection. However, only at 6 h were the differences statistically significant from the wild type (Fig. 5A). Δ*clsA*, Δ*clsB*, and Δ*clsC* mutants showed no significant variation from the wild type, nor did the Δ*clsAC*-mutant genotype (Fig. 5A). In contrast, Δ*clsAB* and Δ*clsBC* were modestly attenuated and, like for the triple mutants, measured significant one-log reductions in CFU at 6 h (Fig. 5A).

The intracellular survival assay involves the use of gentamicin to kill extracellular bacteria. To rule out the possibility that the phenotypes were due to variations in gentamicin sensitivity between the genotypes, we performed MIC tests. Indeed, no significant variation was observed in the gentamicin sensitivity between the mutants and the wild-type strain (Fig. S3). Therefore, *S*. Typhimurium Δ*clsABC*, Δ*clsAB*, and Δ*clsBC* mutants are perturbed for macrophage infection. The data suggest that in the absence of *clsA* and *clsC*, *clsB* is necessary and sufficient to promote infection, while in the absence of *clsB*, *clsA* and *clsC* are necessary and sufficient.

### Macrophages infected with Δ*clsABC*, Δ*clsAB*, and Δ*clsBC* mutants secrete less IL-1β than macrophages infected with wild-type *S*. Typhimurium.

Mitochondrial CL molecules can prime and activate the NLRP3 inflammasome as part of inducing pyroptosis (28). *S*. Typhimurium can activate the inflammasome by multiple mechanisms, and a hallmark of activation is the secretion of the proinflammatory cytokine IL-1β (48, 49). To indirectly measure the contribution of the *cls* enzymes to inflammasome activation by *S*. Typhimurium, we quantified the levels of IL-1β that were secreted into macrophage supernatants at 6 h (Fig. 5B). Consistent with a role for *clsABC* in promoting IL-1β secretion, macrophages infected with the triple mutant measured a significant reduction in the levels of secreted IL-1β relative to those infected with the wild-type bacteria (Fig. 5B). The IL-1β secretion phenotype correlated with the intracellular survival phenotype, since the levels of the secreted cytokine did not significantly differ between the macrophages infected with the wild type and those infected with the Δ*clsA*, Δ*clsB*, Δ*clsC*, or Δ*clsAC* mutants (Fig. 5A and B). Macrophages infected with Δ*clsAB* and Δ*clsBC* double mutants measured statistically reduced amounts of secreted IL-1β relative to those of the wild type, and the levels were identical to those elicited by the infection with Δ*clsABC* triple mutant bacteria (Fig. 5B).

Inflammasome activation is also characterized by increased secretion of IL-18 and increased cell lysis (48). To verify that the observed changes in IL-1β secretion correlated with changes in IL-18 secretion and cell lysis, we quantified the amounts of IL-18 and LDH that were secreted into the supernatants of the infected phagocytes. Indeed, macrophages infected with Δ*clsABC*, Δ*clsAB*, and Δ*clsBC* mutant *S*. Typhimurium secreted less IL-18 and released less LDH than macrophages infected with the wild-type or Δ*clsAC*-mutant bacteria (Fig. S4). Thus, *clsB* is necessary and sufficient to enhance inflammasome activation in the absence of *clsA* and *clsC*, and in the absence of *clsB*, *clsA* and *clsC* are necessary and sufficient.

### Deleting the *cls* genes does not cause wholesale changes in LPS production or affect *S*. Typhimurium’s ability to induce macrophage secretion of TNF-α.

Purified bacterial CL molecules influence TLR4’s ability to detect the lipid A moiety of LPS molecules (23, 26, 50). TNF-α is an inflammatory cytokine that is induced and secreted in response to TLR4 activation (51, 52). Therefore, we sought to determine the contribution of *S*. Typhimurium’s CL synthases to LPS biosynthesis and macrophage secretion of TNF-α. Stationary-phase cultures of wild-type and *cls*-mutant *S*. Typhimurium were normalized to identical cell numbers and subjected to hot-phenol LPS extractions. Compared to that of the wild type, the LPS profile for the single, double, and triple *cls* mutants showed no obvious wholesale differences in LPS abundance with respect to the three length modalities of O-antigen displayed by *S*. Typhimurium: the short, long, or very long modalities. (Fig. 5C). Next, we infected macrophages with wild-type and *cls*-mutant *S*. Typhimurium to determine the levels of TNF-α that were secreted into the cell culture supernatants at 6 h. Macrophages infected with single, double, and triple mutant *S*. Typhimurium secreted statistically identical levels of TNF-α, which suggests that LPS-induced TLR4 signaling is not affected by the absence of the *cls* genes in C57BL/6J mouse macrophages (Fig. 5D).

### Transcomplementation of Δ*clsABC* mutants with the *clsB* or *clsA* operon restores CL biosynthesis but does not restore intracellular survival or IL-1β secretion in macrophages.

**FIG 6:**
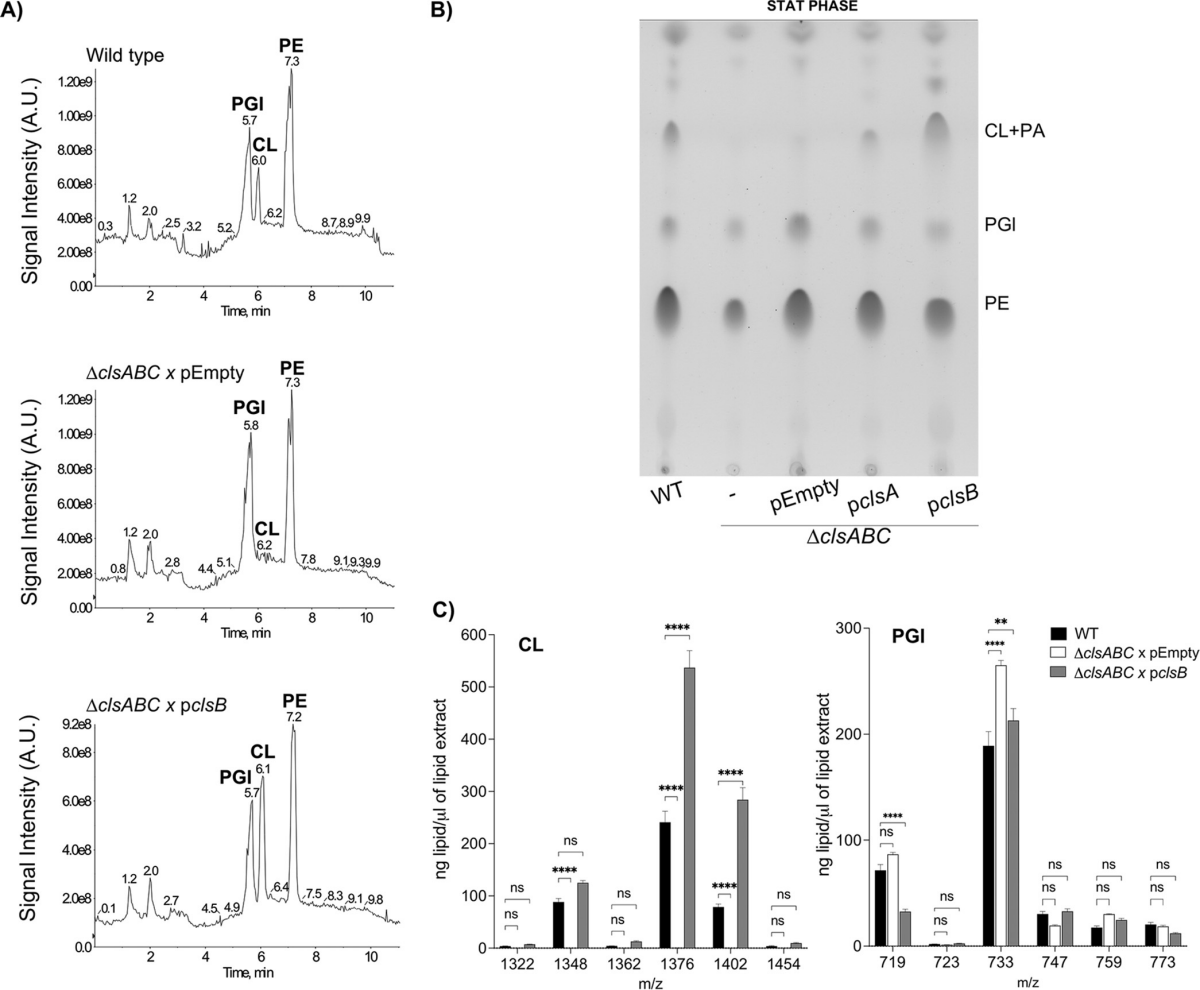
Expressing the *clsB* operon from a plasmid is sufficient to restore CL biosynthesis to the Δ-clsABC mutant. (A) Whole-cell lysates were collected from stationary-phase cultures of the wild type and the Δ*clsABC* mutants expressing an empty vector (pEmpty) and the Δ*clsABC* mutant expressing a plasmid-borne copy of the *clsB* operon (p*clsB*). GPLs were extracted from equivalent amounts of total membrane and were separated and visualized by LC-MS. (B) GPLs from stationary-phase cultures were extracted from total membranes of bacteria and separated by TLC. (C) GPLs were extracted from whole-cell lysates, and the abundance of individual CL and PGl molecules was quantified by LC-MS/MS as the ng of GPL per μL of extract ± SEM. Four biological replicates were analyzed. A two-way ANOVA followed by a Tukey’s multiple-comparison test was used to test significance. Asterisks indicate a significant difference relative to the wild type, ****, *P* < 0.0001; **, *P* < 0.01. ns, not significant.

The phenotypes of the mutants in macrophages were dependent on the combined absence of *clsB* and either *clsA* or *clsC* (Fig. 5A). Since the synthetic phenotypes were partly dependent on *clsB*, we cloned the *clsB* operon into a medium-to-low-copy plasmid vector and introduced it into Δ*clsABC S*. Typhimurium. Adding the *clsB* operon on this plasmid to the Δ*clsABC*-mutant *S*. Typhimurium was sufficient to restore CL biosynthesis and resulted in overproduction of CL relative to that of the wild-type bacteria (Fig. 6A to C).

**FIG 7:**
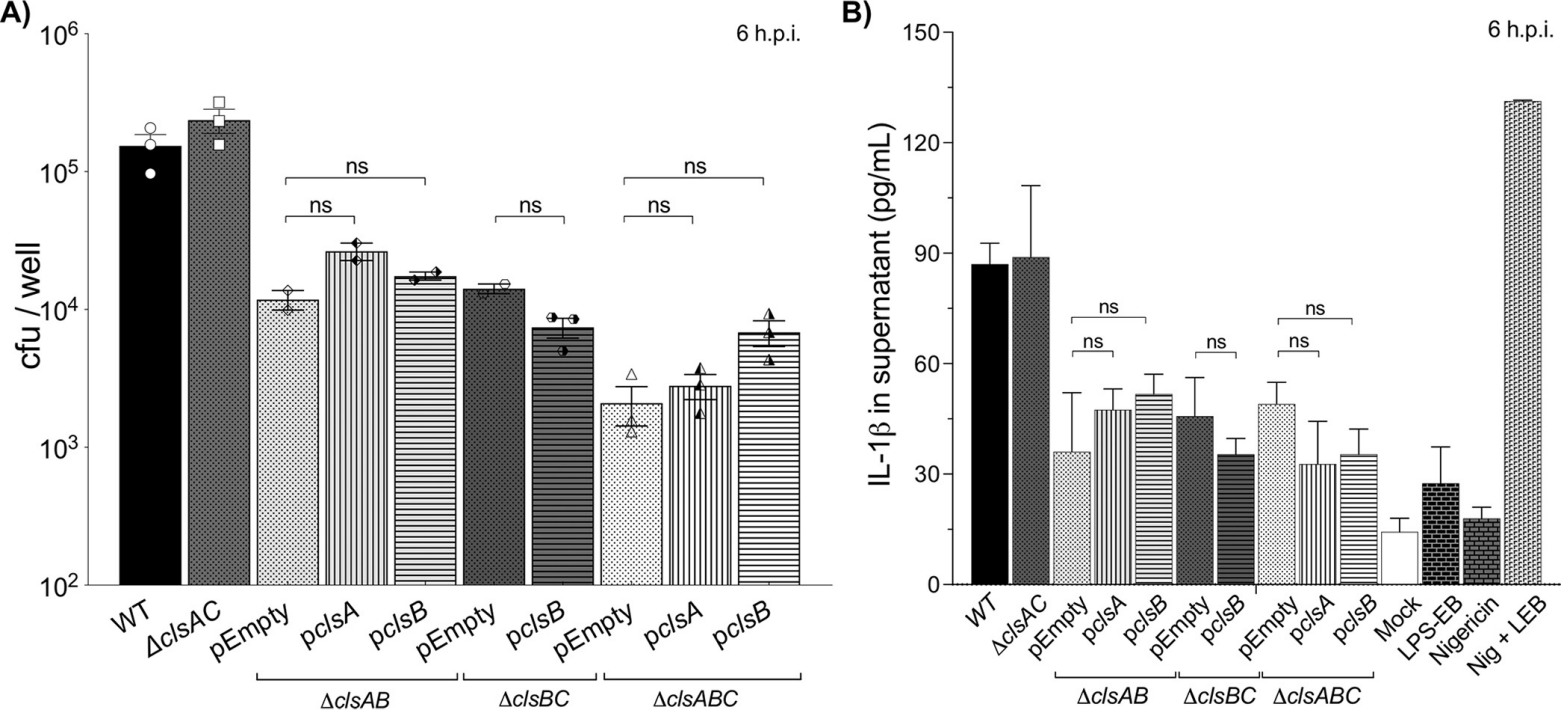
Transcomplementation of Δ*clsAB*-, Δ*clsBC*-, or Δ*clsABC*-mutant *S*. Typhimurium with plasmid-borne *clsA* or *clsB* does not restore the intracellular survival or IL-1β secretion phenotypes in macrophages. (A) Macrophages were infected and lysed at 6 hpi, and the surviving intracellular bacteria were enumerated. Triplicate wells were infected for each genotype. The graph depicts the individual mean number of CFU per well ± SEM for two or three independent experiments, depending on the genotype. Statistical significance was calculated using a one-way ANOVA followed by Dunnett’s posttest. No significant (ns) difference was observed between the complementation strains and pEmpty for each genotype. (B) The supernatants of the infected cells depicted in panel A were collected at 6 hpi, and the levels of secreted IL-1β secretion were freshly quantified using a sandwich ELISA. Three wells were infected per bacterial genotype, and the data are shown as the average pg/mL ± SD of secreted IL-1β. This graph represents one of two or three independent experiments depending on the genotype. Statistical significance was calculated using a one-way ANOVA followed by Dunnett’s posttest. No significant (ns) difference was observed between the complementation strains and pEmpty for each genotype.

We then examined whether the ClsB-dependent CL production could restore the intracellular survival and inflammasome activation defects of the mutants. Surprisingly, expressing *clsB* in *trans* was not sufficient to restore the macrophage infection and IL-1β secretion phenotypes of the Δ*clsAB-*, Δ*clsBC-*, or Δ*clsABC*-mutant *S*. Typhimurium (Fig. 7, Fig. S4). Since it was possible that the *clsB*-dependent defect was unrelated to the catalytic activity of ClsB, we also tested whether expressing a catalytically inactive mutant of ClsB was sufficient to restore intracellular survival to the triple mutant. By TLC analysis, the *clsB^H290A^* mutant gene was catalytically inert and defective for restoring CL production compared to the wild-type *clsB* gene (Fig. S5A). However, expressing the plasmid-borne copy of the catalytically inactive *clsB* gene did not restore the intracellular survival defect of the Δ*clsABC* mutant in macrophages (Fig. S5B). We also attempted to complement the Δ*clsABC* and Δ*clsAB* mutants by expressing the *clsA* operon in *trans*. Indeed, the plasmid-borne copy of *clsA* was sufficient to restore CL production in the triple mutant (Fig. 6B and Fig. S6). However, expressing *clsA* in *trans* still did not rescue the intracellular survival and cytokine secretion defects of the Δ*clsABC* or Δ*clsAB* mutants (Fig. 7A to B). Therefore, the phenotypes caused by inactivating *clsB* in strains that already lack *clsA* (Δ*clsAB*), *clsC* (Δ*clsBC*), or both (Δ*clsABC*) are not attributable to *clsB*-dependent changes in CL content.

## DISCUSSION

*S*. Typhimurium regulates the abundance and structure of the GPL and LPS molecules within the OM in response to fluctuations in the host environment. The conditions in macrophage phagolysosomes elicit bacteria to increase their OM-CL content, but whether these changes affect bacterial pathogenesis had not been formally examined (5, 14, 41, 42). In the current study, we used genetic and biochemical approaches to investigate whether the genes encoding the enzymes that are responsible for CL biosynthesis in *S*. Typhimurium are important for bacterial infection in mice and for the inflammatory response of macrophages to the intracellular pathogen. Our findings establish that, like other Gram-negative enterobacterial microbes, *S*. Typhimurium encodes three phospholipase-D enzymes that collectively contribute to the CL pool (Fig. 1A). As observed in Escherichia coli and Shigella flexneri, ClsA is the primary CL synthase in the log phase of growth, while ClsB and ClsC contribute to CL production in stationary phase (Fig. 2A). Consistent with a nonessential role for CL biosynthesis in this microbe, deleting all three enzymes had no impact on bacterial viability. Adding back the *clsA* or *clsB* operons on a plasmid to the Δ*clsABC* mutants was sufficient to restore CL production to *S*. Typhimurium, and expressing the *clsB* operon resulted in CL overproduction (Fig. 6). Our ability to genetically deplete CL from *S*. Typhimurium inspired us to test the contribution of *clsABC*-dependent CL production to bacterial pathogenesis. Surprisingly, in both systemic and gastrointestinal C57BL/6J mouse models of disease, Δ*clsABC* remained highly virulent (Fig. 3 and 4). We conclude that *clsABC*-dependent CL production is largely dispensable for *S*. Typhimurium pathogenesis in these murine models of infection.

The exact biological function of CL molecules for enterobacterial microorganisms has been elusive, in part due to presence of multiple enzymes capable of CL biosynthesis. The Δ*clsABC* mutant was first generated in a laboratory strain of Escherichia coli and subsequently engineered in Shigella flexneri (16, 17, 20, 53). Phenotypic analyses of Δ*clsABC* mutants in E. coli revealed pleiotropic effects on cell size, cell envelope stress response, surface adhesion, and LPS structure (54). Work in S. flexneri suggested a role for ClsA-mediated CL biosynthesis in promoting intracellular growth and cell-to-cell spread in human epithelial cells (53, 55). A variety of E. coli transmembrane proteins also interact with CL, and CL influences protein function (56–61). In the case of *S*. Typhimurium, *clsABC*-dependent CL biosynthesis is largely dispensable for virulence in C57BL/6J mice and had no obvious effect on LPS composition or abundance (Fig. 3 to 4 and 5C). We reason that other *S*. Typhimurium GPLs might be sufficient to compensate for the loss of CL. The most likely candidate is PGl, which is the principal precursor to CL molecules in bacteria (19). PGl biosynthesis is essential in enterobacterial microorganisms (62). Since the level of PGl molecules is elevated in *S*. Typhimurium Δ*clsABC* mutants relative to that in the wild type, it is conceivable that PGls functionally replace CLs in the context of this triple deletion mutant (Fig. 2C). Alternatively, *S*. Typhimurium might carry an additional enzyme(s), other than ClsABC, which is capable of CL biosynthesis and specifically active in the host environment.

The C57BL/6J inbred mouse model of disease has been used extensively to characterize the pathogenesis of *S*. Typhimurium. However, the model has limitations and caveats. Notably, this mouse genotype carries a mutation in Nramp1 (Slc11a1), a proton-coupled divalent cation transporter, which results in a permissive infection phenotype for *S*. Typhimurium (63, 64). Perhaps ClsABC-dependent CL biosynthesis is necessary for *S*. Typhimurium pathogenesis in Nramp(+/+) mice or outbred animals. Alternatively, ClsABC-dependent CL biosynthesis might contribute to *S*. Typhimurium infection for humans or animals other than mice.

We also examined the contribution of *clsABC* to *S*. Typhimurium intracellular survival in primary C57BL/6J mouse macrophages and measured the cytokine response of phagocytes that were infected with bacterial mutants that lacked one, two, or all three phospholipase-D enzymes. Relative to the wild-type *S*. Typhimurium, the Δ*clsAB*, Δ*clsBC*, and Δ*clsABC* mutants were modestly attenuated for survival in macrophages, while Δ*clsA*, Δ*clsB*, Δ*clsC*, and Δ*clsAC* mutants were not (Fig. 5A). Furthermore, macrophages infected with the wild type and the Δ*clsA*, Δ*clsB*, Δ*clsC*, and Δ*clsAC* mutants secreted identical amounts of IL-1β, IL-18, and LDH, while phagocytes infected with the Δ*clsAB*, Δ*clsBC*, and Δ*clsABC* mutants secreted reduced levels (Fig. 5B, see Fig. S4 in the supplemental material). The genetic data suggested that *clsB* is necessary and sufficient in the absence of *clsA* and *clsC* to promote intracellular survival and inflammasome activation in C57BL/6J mouse macrophages. Likewise, *clsA* and *clsC* are necessary and sufficient in the absence of *clsB*.

Transcomplementation of the Δ*clsABC* mutant with either the *clsA* or *clsB* operons restored CL biosynthesis, which indicated that plasmid-borne enzymes were catalytically active (Fig. 6, Fig. S6). However, expressing these genes in the triple mutant did not rescue the intracellular survival or IL-1β secretion phenotypes (Fig. 7). We rationalized that perhaps failure to restore the phenotype was due to the requirement of a second intact synthase. Therefore, we attempted to complement the double-deletion mutants but were still unsuccessful (Fig. 7). Δ*clsAB* and Δ*clsBC* mutants produce measurable amounts of CL, while Δ*clsABC* mutants are devoid of CL content (Fig. S1B and C). Regardless of their varying CL content, these three genotypes exhibit identical phenotypes in macrophages (Fig. 5A and B). The data suggest that the mutant phenotypes are not caused by a change in CL abundance.

It is possible that Cls enzymes have additional roles independent of CL synthesis, which are needed for intracellular survival and inflammasome activation. Recent work supports a role for ClsB beyond CL biosynthesis in enterobacterial microorganisms (22). ClsB is a promiscuous enzyme capable of synthesizing a variety of GPLs other than CL (18, 21). *S*. Typhimurium, Salmonella enterica serovar Typhi, Salmonella enterica serovar Enteritidis, and Salmonella enterica serovar Paratyphi require ClsB for the production of 6-phosphatidyltrehalose (PT) and 6,6′-diphosphatidyltrehalose (diPT) (22). These two glycophospholipids activate the C-type lectin receptor, known as Mincle/CLEC4E, which is a microbial pattern recognition receptor, resulting in NLRP3 activation and secretion of IL-1β (65, 66). Therefore, ClsB-dependent PT and diPT synthesis might stimulate Mincle and increase the activity of the NLRP3 inflammasome. A Δ*clsB* mutant does not have a phenotype, yet Δ*clsAB* and Δc*lsBC* mutants are attenuated for inflammasome induction (Fig. 5). Thus, ClsB-dependent PT and diPT biosynthesis might proceed preferentially when *clsA* and/or *clsC* are downregulated or deleted. In the same sense, our results also suggest that in Δ*clsB*, *clsA* or *clsC* would compensate for the functionality of *clsB*, possibly activating the inflammasome independently of PT or diPT. In this work, we did not verify if these other ClsB-dependent molecules were synthesized in the transcomplemented strains. Likewise, we did not deduce the role of ClsA or ClsC that is sufficient to activate the inflammasome in the absence of *clsB*.

Alterations in the structure and amount of bacterial LPS molecules can also influence inflammasome activation (67–69). Although we did not observe any drastic variation in the abundance of LPS molecules between the genotypes, we cannot rule out that deleting combinations of the *cls* genes causes a minor difference in the total amount of LPS or a structural alteration in the lipid A moiety that reduces inflammasome activation (Fig. 5C). Our failure to complement the mutant phenotypes in macrophages suggests that deleting the *cls* genes themselves might not have a direct consequence. Rather, it is possible that in the process of generating the Δ*clsAB*, Δc*lsBC*, and Δ*clsABC* mutants, a common second-site mutation occurs and causes the attenuation. Future genetic analysis will be necessary to test this prediction.

The primary goal of our study was to understand the role of *S*. Typhimurium’s three CL synthases during pathogenesis for mice and to assess the contribution of bacterial CL biosynthesis to infection-induced inflammasome activation in mouse macrophages. We present strong genetic evidence to support that *clsABC*-dependent CL production is largely dispensable for bacterial virulence in C57BL/6J mice and has no direct role in the ability of *S*. Typhimurium to activate the inflammasome of C57BL/6J mouse macrophages. Although we identified the synthetic role for *clsB* in promoting intracellular survival and inflammasome induction, our genetic analysis supports that the phenotype is not necessarily related to CL biosynthesis and possibly the result an unknown second-site mutation. Additional studies will be necessary to determine the exact cause of the synthetic defect and to assess whether the Cls enzymes influence *S*. Typhimurium pathogenesis in other disease models.

## MATERIALS AND METHODS

### Ethics statement.

All animal procedures were carried out with approval from the University of Oklahoma Health Sciences Center Institutional Animal Care and Use Committee under protocol number 19-015-ACI. The procedures used in this study strictly adhered to the guidelines found in the National Research Council’s Guide for the Care and Use of Laboratory Animals (70).

### Bacterial strains and culturing conditions.

The bacterial strains used in this study were derivatives of the Salmonella enterica serovar Typhimurium genotype 14028s (Table 1). Each strain was streaked onto Luria-Bertani (LB; Difco) agar plates, also known as lysogeny broth, and incubated aerobically at 37°C overnight. Colonies were isolated from −80°C glycerol stocks, weekly. A single colony was typically inoculated into LB broth medium and shaken or rotated at 250 rpm aerobically at 37°C until the logarithmic (log) growth phase, which was defined as an optical density at 600 nm (OD_600_) of 0.6 to 0.8, or the stationary (stat) growth phase, which we define as 16 h post-single colony inoculation. Culturing volumes varied depending on the phenotypic assay: for macrophage and mouse infections, 5 mL culture volumes were used. To collect membranes for glycerophospholipid (GPL) quantification, 1 L cultures were used. The specific details are given in the individual assay description.

### Bacterial genetics.

All strains and primers are listed in Tables 1 and 2, respectively. The STM14_2103 (*clsA::tetRA*) deletion-insertion genotype, which encodes tetracycline resistance, was constructed as part of this study using the phage lambda-red recombinase system (71). The deletion-insertion allele was then horizontally transferred into a fresh nonpKD46-bearing wild-type *S*. Typhimurium genotype using bacteriophage P22 HT105/1 int-201. The other *cls* mutants were generated in our wild-type *S*. Typhimurium 14028s recipient genotype by successive P22 phage transductions using donor genotypes that carry lambda-red directed deletion-insertion alleles in STM14_0942 (*clsB::kan* or *clsB::cat*) and STM14_1314 (*clsC::cat* or *clsC::kan*) (72). To generate the complementation vectors, the *clsA* and *clsB* operons were amplified from the chromosome of *S*. Typhimurium 14028s. The *clsA* operon was cloned into the pWSK29 vector using Gibson Assembly. The *clsB* operon was digested with BstXI and XbaI restriction enzymes and cloned into a BstXI/XbaI digest of pWSK29. The ligated vectors were then electroporated into an E. coli DH5-α expression genotype. The pWSK29-*cls* plasmids were subsequently purified and electroporated into the corresponding *S*. Typhimurium *cls*-mutant genotypes. All deletion genotypes were confirmed by PCR using a forward primer designed to anneal outside the target deletion site on the chromosome and a reverse primer designed to anneal within the resistance cassette, which had replaced the *cls* gene sequence. Deletions of *clsB* and *clsC* were also confirmed by sequencing using a primer that annealed upstream of deletion-insertion site. Complementation plasmids were confirmed by PCR using primer combinations that annealed to a site that was internal to the *clsA* or *clsB* operon and to a site that was external to the operon and within the backbone of the plasmid. The plasmid carrying the *clsB* operon, pWSK29-*clsB*, was used as a template for a PCR to generate the *clsB^H290A^* mutant allele. The primers used in the reaction contained the codon for the desired substitution. Plasmids that contained the mutation were confirmed by sequencing using a primer that annealed upstream of the mutant codon.

**TABLE 2:** Primers used in this study

Use	Primer name	Primer sequence (5′→3′)
To generate Δ*clsA::tetRA*	Fwd clsA-tetRA	AAACTCTTAACAACACGCTTTCTAAAGGATTTTTAAAGTT TTAAGAACCCACTTTCACA
To generate Δ*clsA::tetRA*	Rvs clsA-tetRA	AAACTCTTAACAACACGCTTTCTAAAGGATTTTTAAAGTT TTAAGAACCCACTTTCACA
To generate pWSK*clsAoperon*	GibsA_clsA-pWSK Fwd	GACGGTATCGATAAGCTTGATATCGAATTCatgcgcaggtttcggtcaaac
To generate pWSK*clsAoperon*	GibsC_pWSK-clsA Rvs	gtttgaccgaaacctgcgcatGAATTCGATATCAAGCTTATCGATACCGTC
To generate pWSK*clsAoperon*	GibsD_pWSK-clsA Fwd	gtctgattgcgccctccgCTGCAGCCCGGGGGAT
To generate pWSK*clsAoperon*	GibsB_clsA-pWSK Rvs	ATCCCCCGGGCTGCAGcggagggcgcaatcagac
To generate pWSK*clsB^H290A^*	ClsB_H290A_Fwd	GCGCCACTTTGCCAGCCAGCGGACGTCGTC
To generate pWSK*clsB^H290A^*	ClsB_H290A_Rev	GACGACGTCCGCTGGCTGGCAAAGTGGCGC
For sequencing pWSK*clsB^H290A^*	clsB_chk_H290A_F	CGCCTCTACTGGGGTTGC
For *clsB* confirmation (PCR and seq)	Fwd_clsB_OUT	GGAGGAGATATTCACCCGTG
For *clsB* confirmation (PCR and seq)	Rev_clsB_OUT	ATAAACCACCAGCAGAACGAC
For *clsC* confirmation (PCR and seq)	Fwd_clsC_OUT	ATACCGGAATTGTTTGCTGC
For *clsC* confirmation (PCR and seq)	Rev_clsC_OUT	TATATTACGCCGCATATCTCCT
For km_ confirmation	Rev_km_cassette	CTTCTTGACGAGTTCTTCTGA
For cat_cassette confirmation	Rev_cat_cassette	CACTCATCGCAGTACTGTTGTAAT
For tetRA_cassette confirmation	Rev_tet_internal	TGATAATACAGATACCGAA
To generate pWSK*clsBoperon*	clsB operon 5′ promoter base 880226	CACCTGACCATCGCGTTGGACCGCATCTTTTTGTGACGACGACTACACTATT
To generate pWSK*clsBoperon*	clsB_operon_end base 877202	CTATTCATCTAGATTATTTCGCCATCGCCCGCT

### Harvesting whole-cell lysates from *S*. Typhimurium.

Stationary-phase cultures were normalized to an OD_600_ of 0.7 and harvested by centrifugation. The pellets were resuspended in 25 mL of a solution containing 0.2 M sucrose, 10 mM Tris (pH 7.5), 2.2 μM MgCl_2_, 1 μL of RNase/DNase nuclease reagent, and 1 μL of protease inhibitor cocktail and subjected to pressurized homogenization and lysis using an Emulsiflex-C3 (Avestin). The samples were measured for total proteins, processed for lipid extraction, and stored at −20°C.

### Isolating total membranes from *S*. Typhimurium.

Total membranes were collected and separated as described in reference 5. Briefly, stationary-phase cultures were normalized to an OD_600_ of 0.7 and harvested by centrifugation. The pellets were resuspended in a solution of 0.5 M sucrose (Sigma no. S0389) and 10 mM Tris (pH 7.5; JTBaker no. 4109-6). Lysozyme (144 μg/mL; Alfa Aesar no. J60701) and EDTA (1.5 mM; Sigma-Aldrich no. E9884) were added, and the suspensions were centrifuged. The pellets were resuspended in a solution of 0.2 M sucrose and 10 mM Tris (pH 7.5). After a pressurized homogenization and lysis, samples were ultracentrifuged at 184,500 × *g* for at least 1 h at 4°C. Total membranes were resuspended in 10 mM Tris buffer (pH 7.5) and stored at −20°C.

### Protein quantification and glycerophospholipid extraction.

Protein concentrations were measured using Pierce Coomassie Plus Bradford assay reagent (Thermo Scientific no. 23238) and a standard curve, which was generated from bovine serum albumin standard (BSA; VWR no. 0332). To extract GPLs from the total membrane fractions and whole-cell lysates prior to mass spectrometry, an equivalence of 1 mg of protein for each membrane was subjected to the Bligh-Dyer method (73). Briefly, the corresponding volume of sample equivalent to 1 mg of protein was added to an empty 15 mL polypropylene tube. The necessary amount of water (VWR 02-0201) was added to complete 500 μL of the aqueous phase. Subsequently, methanol (Fischer Chemicals no. A452SK-4) and chloroform (Fisher Chemical no. C607-4) were added to first generate the single-phase mixture. After incubation, a two-phase mixture was generated. The mixture was vortexed and centrifuged at 4,500 rpm to partition the organic phase from the aqueous phase. The lower organic phase was harvested by pipetting and was transferred into an appropriate glass vial (VRR no. 66011-020), and the GPLs were dried under nitrogen gas.

### Normal-phase LC-MS/MS.

Samples were delivered to an Applied Biosystems Sciex API 4000 Triple Quad mass spectrometer using a Waters Acquity H-class ultraperformance liquid chromatography (UPLC) system interfaced with an Agilent Zorbax Rx-SIL column (2.1 by 100 mm, 1.8 μm). Analytes were ionized by electrospray in [M-H]^−1^ mode with a voltage of −4.5 kV. The source temperature was 450°C. Nitrogen was used as the curtain gas (setting 10), nebulizer gas (setting 20), and turbo gas (setting 20). MS/MS was performed using nitrogen as the collision gas (setting 4.0). The declustering, entrance, and collision cell exit potential were −120, −10, and −15, respectively. Retention of PGl and CL was achieved at a flow rate of 0.35 mL/min using mobile phase A (CHCl_3_/CH_3_OH/NH_4_OH [800:195:5, vol/vol/vol]) and mobile phase B (CHCl_3_/CH_3_OH/H_2_O/NH_4_OH [600:340:50:5, vol/vol/vol/vol]). A three-step gradient used started at 0% B for 1 min, continued at 0 to 50% B over the next 3 min, was held at 50% B for 4 min, returned to starting conditions in 0.1 min, and was allowed to equilibrate for an additional 3 min, giving a total run time of ~11.1 min. The following parent-to-daughter ion transitions were monitored (multiple reaction monitoring [MRM]): 719 > 255 PGl, 723 > 255 PGl, 733 > 267 PGl, 747 > 255 PGl, 759 > 267 PGl, 773 > 281PGl; 1322 > 253 CL, 1348 > 253 CL, 1376 > 281 CL, 1402 > 281 CL, 1454 > 281 CL. In the case of PGl molecules, singly charged [M-H]^−1^ parent > daughter ion transitions were monitored. For CL, doubly charged [M-2H]^−2^ parent ions were monitored because they were more abundant than singly charged ions. Collision energies ranged from −35 to −40V for the individual analytes. The data were acquired with the Sciex Analyst software version 1.6.2 (Applied Biosystems, Foster City, CA, USA).

The Bligh-Dyer phospholipid extracts from the membranes were dried under N_2_ gas and resuspended in 300 μL of mobile phase A. A total of 60 μL was loaded into glass inserts within sample vials and added to the sample manager for the UPLC console. The flow through needle delivered 10 μL injections for each membrane GPL extract over the column. The retention times for the major GPL families were confirmed and standard curves were generated using commercial standard from Avanti Polar lipids. These included 1-palmitoyl-2-oleoyl-sn-glycero-3-phospho-(1′-rac-glycerol), (C16:0, C18:1) PGl, *m/z* 747.6, and 1′,3′-bis[1-palmitoyl-2-oleoyl-sn-glycero-3-phospho]-glycerol, (C16:0, C18:1, C16:0, C18:1) [M-H]^−1^ CL *m/z* 1,404 or [M-2H]^−2^ *m/z* 702. Integrated peak areas were plotted against standard concentration to generate a linear equation to which the peak area values generated from specific PGl and CL parent > daughter ion transitions were applied. Using our standard curve, we calculated the amount (ng) of the PGl and CL per μL of sample. At least four independent biological replicates per strain were examined, quantified, and averaged to give the ng/μL concentration for each individual GPL species within the extract.

### TLC.

An equivalence of 2 mg of protein for each membrane was extracted by Bligh-Dyer method (73). The chloroform layer was harvested and dried under nitrogen gas, and the GPLs were resuspended in 60 μL of chloroform (Fisher Chemical no. C607-4). TLC was performed on silica gel 60 plates (Sigma no. 1057210001). The GPLs were spotted as successive 20 μL droplets onto the origin of the TLC plate and dried. For one-dimensional TLC, a mobile phase of chloroform/methanol/acetic acid (130:50:20) was used. In case of two-dimensional TLC, a mobile phase of chloroform/methanol/water (120:50:8) was used for the first dimension, and a mobile phase of chloroform/methanol/acetic acid/water (160:24:30:8) was used for the second dimension. The GPLs were visualized by iodine vapor and identified using commercial standards CL (no. 710341), PGl (no. 840457), PE (no. 850757), and PA (no. 840857) (Avanti).

### Intraperitoneal infections in mice.

Male and female C57BL/6J mice were purchased from Jackson Laboratories and bred in-house under pathogen-free conditions. To measure the ability of *S*. Typhimurium to survive systemically and colonize the spleens and livers of mice, 6- to 8-week-old mice were intraperitoneally (i.p.) infected with roughly 5 × 10^5^ CFU. *S*. Typhimurium was grown aerobically overnight in LB, pelleted, resuspended in phosphate-buffered saline (PBS; Corning no. 21-040-CV), and diluted to an appropriate concentration based on the OD_600_ of the solution of cells. The inoculum was administered i.p. in a single 200 μL bolus. Serially diluting the inoculum and plating onto LB plates verified the actual number of CFU that were administered. At 48 h postinfection (hpi), the mice were euthanized and the livers and spleens were dissected, weighed, and homogenized in PBS-Triton X-100 0.1% (Merk-Millipore no. TX1568-1). Organ homogenates were serially diluted and plated onto LB agar and incubated overnight at 37°C. The CFU counts were normalized to the individual organ weight. For the murine lethality studies and time-to-death experiments, 6- to 8-week-old mice were i.p. infected with roughly 5 × 10^3^ CFU The weight, appearance, and behavior of each infected animal were monitored daily using a standard mouse pain, distress, and morbidity scoring system. Measurements were taken until at least 21 days postinfection or until mice achieved a threshold morbidity score. At day 21, the livers and spleens of the deceased mice were dissected, weighed, homogenized, and plated to enumerate CFU.

### Gastrointestinal mouse infections.

For oral *S*. Typhimurium infections, mice were orally gavaged with 20 mg/mouse of streptomycin (Sigma no. S-6501) solubilized in PBS. Twenty-four hours later, the streptomycin-treated mice were orally gavaged with 1 × 10^8^ CFU of bacteria that were resuspended in 200 μL of PBS. The dose was verified by plating serial dilutions of the bacterial suspension onto LB agar plates. Fecal samples were collected daily for five consecutive days. The feces were weighed, serially diluted in PBS, and plated onto MacConkey agar plates to enumerate the CFU/g of feces. For the lethality assay, the streptomycin-treated mice were orally gavaged with 1 × 10^5^ CFU of bacteria in 200 μL of PBS. The weight, appearance, and behavior of each infected animal were monitored daily using a standard mouse pain, distress, and morbidity scoring system. Measurements were taken until mice achieved a threshold morbidity score, and then mice were sacrificed.

### Differentiation of murine macrophages.

Primary bone marrow-derived murine macrophages (BMDMs) were prepared by harvesting the marrow from the femurs of 6- to 8-week-old C57BL/6 mice from Jackson Laboratories. Macrophage colony-stimulating factor (M-CSF) was prepared from the supernatants of stably transfected NIH 3T3 immortalized fibroblast cells. The macrophages were differentiated at 37°C with 5% CO_2_, in BMDM medium, which consisted of RPMI 1640 with 20% fetal bovine serum (FBS), 10% M-CSF, 1% l-glutamine, 1% sodium pyruvate, 1% penicillin-streptomycin, and 50 μM β-mercaptoethanol (OmniPur no. 6010). After 1 week, macrophages were replated at 2.5 × 10^5^ cells/well in 24-well plates.

### Intracellular survival in murine macrophages.

Macrophages were seeded at 2.5 × 10^5^ cells per well in 24-well plates. After attachment to the wells, the cells were primed using interferon gamma (INFγ; carrier-free; 150 U/mL; Biolegend no. 57308) for 16 h prior to the bacterial infections. The macrophages were infected with stationary-phase salmonellae at a multiplicity of infection (MOI) of 10:1 by centrifugation for 10 min at 4°C. The infectious dose was corroborated by serial dilutions and plating, aiming to infect the macrophages with the same initial CFU. For all the experiments that involved infection of macrophages, the initial CFU was not statistically different between strains across the independent biological replicates. Infected macrophages were incubated at 37°C under 5% CO_2_ for 1 h and washed three times with PBS to remove extracellular bacteria. 250 μL of RPMI + FBS with 100 μg/mL of gentamicin sulfate (VWR no. 0304) was added to the wells to be collected at 2 hpi, and 10 μg/mL of gentamicin was added to the wells to be collected at 6 hpi At the corresponding time, cells were washed with PBS, and PBS + 0.1% Triton X-100 was added for macrophage lysis, and monolayers were gently scraped and collected. Three wells per bacterial genotype were assessed per time point. Surviving intracellular CFU were enumerated by plating serial dilutions.

### Quantification of secreted cytokines and lactate dehydrogenase from infected macrophages.

The intracellular survival assay and cytokine measurements were done concomitantly, using the same infected cells. That is, 250 μL of the supernatant was harvested to measure secreted proteins, and the adhered macrophages were lysed for CFU counts as described for the intracellular survival assay. Enzyme-linked immunosorbent assays (ELISA) were used to quantify the murine cytokines IL-1β, IL-18, and TNF-α from cell-free supernatants. For IL-1β, the ELISA coating and detection antibodies were selective for the proteolytically processed form of the mouse cytokine (clone B122, purified, eBioscience, no. 14-7012-85 and polyclonal, biotinylated, eBioscience, no. 13-7112-85). For TNF-α, the coating and detection antibodies were clone 1F3FD4, purified, eBioscience, no. 14-7325-85 and MP6-XT3 & MP6-XT22, biotinylated, eBioscience, no. 13-7326-85, respectively. As a control of TNF-α secretion, macrophages were treated with 10 ng/mL lipopolysaccharide from Escherichia coli O111:B4 (LPS-EB; InVivoGen no. tlr-eblps) for 6 h. As a control for IL-1β secretion, 10 μM nigericin (ENZO no. BML-CA421-0005) alone or in combination with LPS-EB was used. IL-18 was measured using mouse IL-18 ELISA pair set (Sinobiological Inc. no. SEK50073) following manufacture’s guidelines. The cytokines were measured on the day of harvest, from triplicate wells. A calibration curve was generated using commercial standards: mouse IL-1β ELISA standard (Thermo Scientific no. 29-8012-65) and mouse TNF-α ELISA standard (Thermo Scientific no. 29-8321-65). Standards were diluted in 1% BSA (VWR no. 0332) in PBS. For the TNF-α measurements, the supernatants were diluted 1:50 in 1% BSA (VWR no. 0332) in PBS. For the IL-1β and IL-18 measurements, the supernatants were not diluted.

Cell death was determined by measuring the amount of lactate dehydrogenase that was secreted into the cell-free supernatants using the CyQUANT LDH cytotoxicity assay (Thermo Scientific no. C20301) kit according to the protocol. The LDH was measured in triplicate wells.

### LPS extraction, electrophoresis, and detection.

Stationary-phase bacterial cultures (5 mL) were normalized to an OD_600_ of 2.0, pelleted, and resuspended in 200 μL of diethyl pyrocarbonate (DEPC)-treated endotoxin-free water (GBiosciences no. 786-109). To corroborate that each sample contained approximately the same CFU level, 20 μL of the 200 μL resuspension was used for serial dilutions and plated to enumerate the real number of bacteria in the solution. Next, 2 μL of 2% SDS was added to the remaining 180 μL of sample and the solution was boiled for 5 min. Five microliters of proteinase K (New English BioLabs no. P8107S) was added to the reaction, and the samples were incubated at 59°C overnight. Ice-cold Tris-saturated phenol (Sigma no. 4557) was added (1:1), and the samples were vortexed and incubated at 70°C for 15 min. After cooling, the solution was centrifuged and a two-phase solution was observed. The top layer was harvested. Samples were mixed with Laemmli sample buffer 4× (Bio-Rad), and 15-μL aliquots were separated by denaturing gel electrophoresis using 4 to 15% polyacrylamide gradient gels (Tris-glycine-SDS buffer; Bio-Rad) and stained with ProQ300Emerald lipopolysaccharide gel staining kit (Molecular Probes) following the manufacturer’s instruction. Pictures of gels were taken with ChemiDoc MP imaging system (BIORAD), and the images were analyzed by Image Lab 6.0 Software (BIORAD).

### MIC.

To quantify the MIC of gentamicin for the *S*. Typhimurium genotypes, wild type, Δ*clsAB*, Δ*clsAC*, Δ*clsBC*, and Δ*clsABC* were grown in LB broth to the stationary phase and normalized to an OD_600_ of 0.1 (which approximately corresponds to 0.5 McFarland, or 1 × 10^8^ CFU/mL). To verify that we were assessing equivalent numbers of bacteria, the bacterial suspensions were serially diluted and plated onto LB to obtain colony counts. A total of 100 μL of the normalized resuspension of bacteria was plated onto LB agar (25 mL per plate). The gentamicin-MIC test strips (Liofilchem no. 92009; testing a range of 0.016 to 256 μg/mL) were placed in the middle of the agar surface, and the plates were inverted and incubated at 37°C for 16 h. Visualizing the intersection between the ellipse of growth inhibition and the strip allowed us to assess the MIC.

### Statistical analysis.

All statistical analyses and graphs were performed using Prism 9 software (GraphPad Software, La Jolla, CA, USA). Unless otherwise stated, numeric data expressed were representative of at least three biological replicates, each one done by triplicate, and presented as mean ± standard error of the mean (SEM). Statistical significance was defined as *P *<* *0.05, (*, *P* < 0.05; **, *P* < 0.01; ***, *P* < 0.001; ****, *P* < 0.0001; no asterisk, not significant).
