# Supplementary material for: Cardiolipin Biosynthesis Genes Are Not Required for Salmonella enterica Serovar Typhimurium Pathogenesis in C57BL/6J Mice
Source: Microbiol Spectr. 2022 May 31;10(3):e02617-21. doi: 10.1128/spectrum.02617-21 (PMC9241728; doi:10.1128/spectrum.02617-21)
Supplement: SUPPLEMENTAL FILE 1 — Supplemental material. Download spectrum.02617-21-s0001.pdf, PDF file, 5.1 MB [file spectrum.02617-21-s0001.pdf]

SUPLEMENTAL FIGURES AND LEGENDS

Fig.S1)

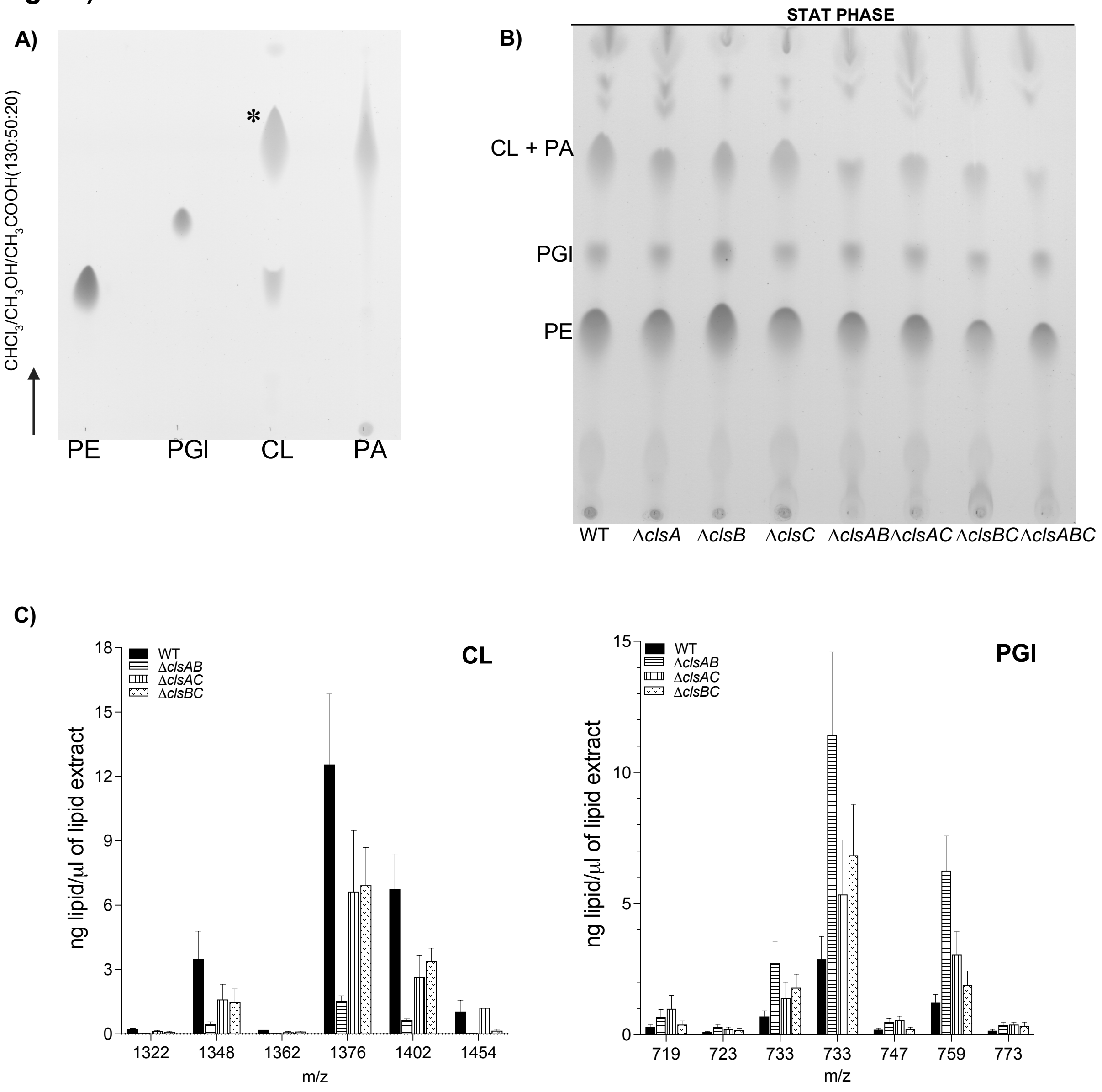

Figure S1. Combined deletion of *clsA*, *clsB*, and *clsC* abrogates cardiolipin (CL) synthesis. A) Commercial standards of phosphatidylethanolamine (PE), phosphatidylglycerol (PG), CL, and phosphatidic acid (PA) were used to determine the retention factors of each of the lipid species in the solvent system used for chromatographic separations. 0.25mg of the lyophilized standards were resuspended in chloroform and then spotted onto the silica thin-layer chromatography (TLC) plate. The

mobile phase consisted of the mixture of chloroform/methanol/acetic acid (130:50:20). Iodine staining was performed followed by digitalization of the image. * The asterisk denotes the spot that corresponds to CL, which we will use in the text for comparison between genotypes. **B)** Total membrane fractions of the wild type, and *cls*-mutants *S.* Typhimurium were harvested at stationary phase of growth. Glycerophospholipids (GPLs) were separated using chloroform/methanol/acetic acid (130:50:20) as the mobile phase for TLC. Iodine staining was performed and the image was digitalized. **C)** Total membrane fractions of the wild-type and *cls*-double mutants *S.* Typhimurium were collected at stationary phase of growth. CL and PGl molecules were quantified by normal-phase liquid-chromatography tandem mass spectrometry (LC-MS/MS). The quantities are presented as the nanograms of lipid per microliter (ng/µl) of extract ± standard error of the mean (SEM). The data reflect the average from four biological replicate. A one-way ANOVA followed by the Bonferroni post-test was used to assess statistical significance. No statistical differences were found between the wild type and *cls*-mutant genotypes.

Fig.S2)

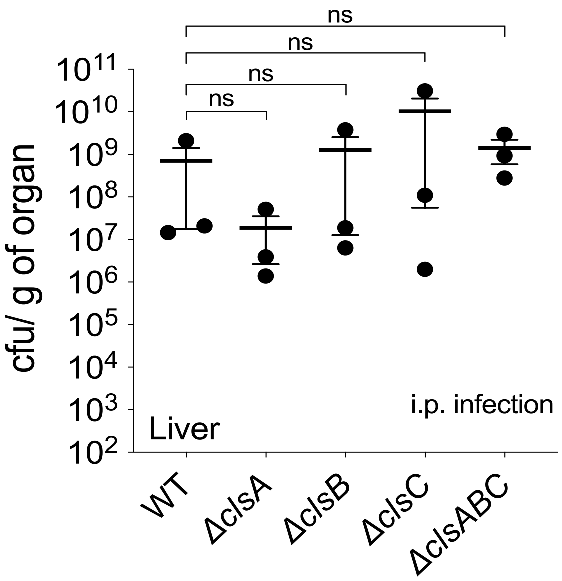

Figure S2. Wild-type, Δ*clsA*, Δ*clsB*, Δ*clsC*, and Δ*clsABC S.* Typhimurium are recovered from the livers of mice at statistically identical levels following intraperitoneal injection. C57BL/6J mice were intraperitoneally (i.p.) injected with 5 x10^5 colony-forming units (cfu) of the wild type, Δ*clsA*, Δ*clsB,* Δ*clsC,* and Δ*clsABC* mutant *S*. Typhimurium. After 2 days post-infection (p.i.), the mice were euthanized and colony counts were enumerated from liver homogenates. Data are shown as the mean number of cfu/g of liver tissue ± SEM. Each genotype was assessed in three mice. A one-way ANOVA was executed to determine significance, and no statistical difference was observed between the mutants and the wild type.

Fig.S3)

Strain	Minimum Inhibitory Concentration (M.I.C.)
WT	3μg/mL
Δ*clsAB*	3μg/mL
Δ*clsAC*	3μg/mL
Δ*clsBC*	3μg/mL
Δ*clsABC*	3μg/mL

Figure S3. Wild type, Δ*clsAB*, Δ*clsBC*, Δ*clsAC*, and Δ*clsABC* are equally sensitive to gentamycin. Gentamycin was added to the macrophage cultures to kill extracellular bacteria. To ensure that the differences in intracellular survival for the Δ*clsAB*, Δ*clsBC*, and Δ*clsABC* mutants were not due to variations in gentamycin sensitivity (**Fig. 5A, 7A**), we performed minimal inhibitory concentration (MIC) tests. Stationary-phase cultures of wild type, Δ*clsAB*, Δ*clsBC*, Δ*clsAC,* and Δ*clsABC* mutant *S.* Typhimurium were normalized to an optical density at 600nm (OD_{600}) of 0.1, to achieve 0.5 McFarland suspensions. 100μL of each suspension was seeded onto Luria-Bertani (LB) (also known as Lysogeny broth) agar plates and a gentamycin test strip was applied onto the surface of each plate. After 16h, the MIC was read. Data in the table represent one of three independent experiments.

Fig.S4)

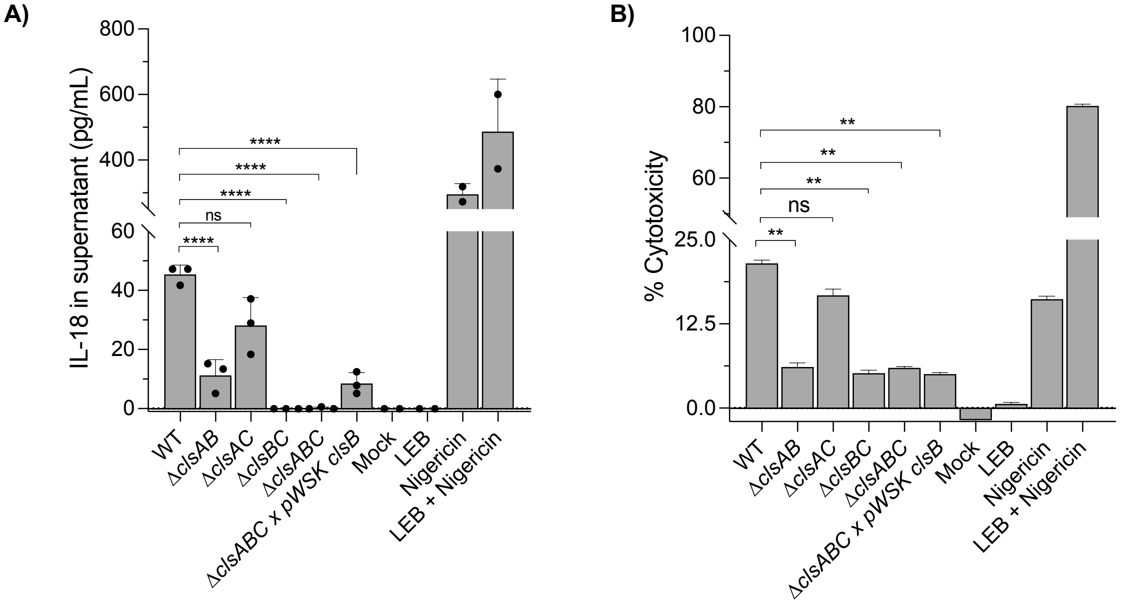

Fig. S4. *S.* Typhimurium strains that lack *clsA* or *clsC* require *clsB* to promote macrophage secretion of interlukin-18 (IL-18) and lactate dehydrogenase (LDH). **A)** Secretion of IL-18 was measured in the cell free supernatants of infected mouse bone marrow derived macrophages (BMDM), which were previously treated with interferon gamma (INFγ) (150 U/ml). Macrophages were infected under the same conditions as in Figures 5 and 7. After six hours post infection (h.p.i), the supernatants were harvested and a sandwich ELISA measured the cytokines. LPS-EB (10 ng/mL), Nigericin (10 μM) and a combination of both were used as controls. Calibration curves were performed using commercial standards. Data are shown as the average pg/ml of IL-18 from triplicate wells of infected BMDMs for each condition. The graph represents one of two independent experiments. A one-way ANOVA followed by Dunnet's post-test was performed to assess statistical significance (****p<0.0001). **B)** Lactate dehydrogenase (LDH) activity was measured in the supernatants of the infected BMDMs at 6 h.p.i. Percentage of cytotoxicity was calculated as the activity of released

LDH relative to total LDH activity (by using a lysis buffer provided by the kit), assayed in triplicate. The graph represents one of two independent experiments. A one-way ANOVA followed by Dunnet’s post-test was performed to assess statistical significance (**$p<0.01$)

Fig. S5.

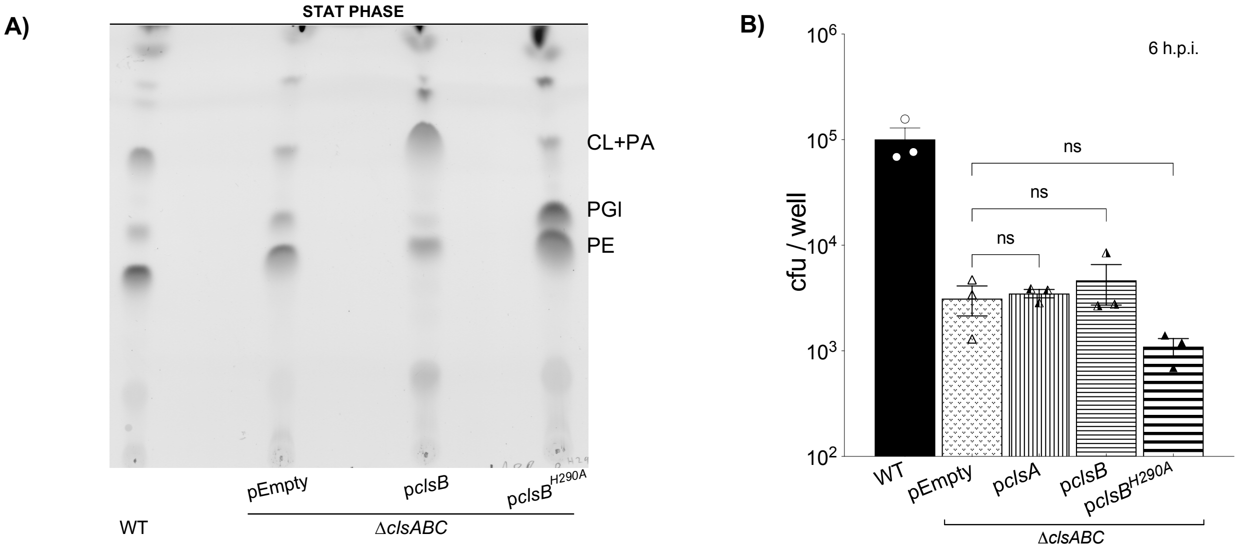

Fig. S5. Transcomplementation of the *ΔclsABC* mutant *S.* Typhimurium with the *clsB* operon restores CL biosynthesis, but not intracellular survival. A) Total membranes were collected from stationary phase cultures of wild-type, and Δ*clsABC* mutants expressing a plasmid borne copy of the empty vector (pEmpty), the *clsB* operon (p*clsB*), or the the *clsB* operon encoding a catalytically inactive ClsB enzyme (p*clsB*^{H290A}). The GPLs were extracted from equivalent amounts of total membranes (2mg). The lipids were separated and visualized by one-dimensional TLC using chloroform/methanol/acetic acid (130:50:20) as the mobile phase **B)** Intracellular survival was assessed in macrophages after infection with wild type, and Δ*clsABC* mutants expressing a plasmid borne copy of the empty vector (*pEmpty*), the *clsA* operon (*pclsA*), *clsB* operon (*pclsB*), *clsB* operon encoding a catalytically inactive ClsB enzyme (*pclsB*^{H290A}). After 6 h.p.i., macrophages were lysed and the intracellular bacteria was serially diluted and plated. The data represents the mean between cfu/well ± (SEM) from three biological replicates. Each of the biological replicates was

performed in triplicate for each strain. Statistical differences were calculated by using One-way ANOVA, followed by Bonferroni post-test. No significant (ns) difference was observed between the mutant genotype carrying the empty vector and the genotypes expressing the plasmid borne copies of *clsA* or *clsB*.

Fig. S6.

A)

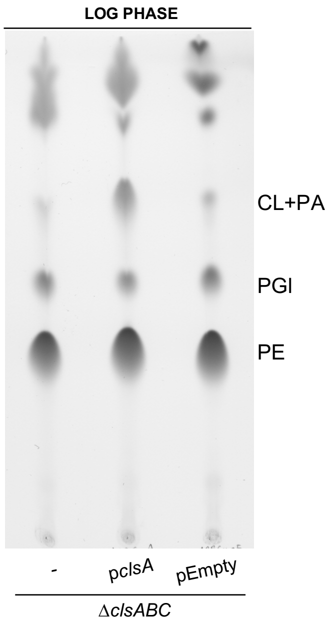

B)

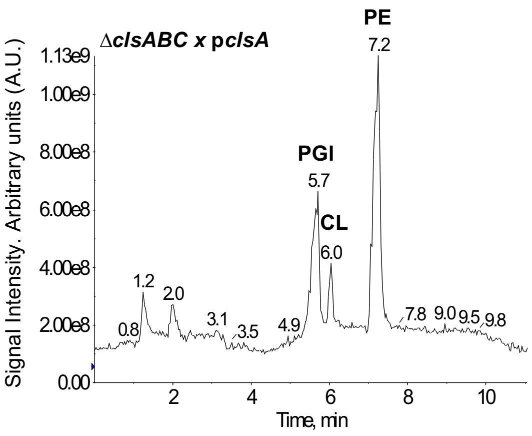

Fig. S6. Transcomplementation of *ΔclsABC* mutant *S.* Typhimurium with the *clsA* operon restores CL biosynthesis. A) Total membranes were collected from logarithmic phase cultures of Δ*clsABC*, and Δ*clsABC* expressing a plasmid borne copy of the *clsA* operon (p*clsA*), and empty vector (p*Empty*). GPLs were extracted from equivalent amounts of total membranes (2mg The lipids were separated and visualized by one-dimensional TLC using chloroform/methanol/acetic acid (130:50:20) as the mobile phase. **B)** Total membranes were collected from from stationary phase cultures of *ΔclsABC* that expressed a plasmid borne copy of the *clsA* operon (p*clsA*). GPLs were extracted from equivalent amounts of total membrane (1mg) and were separated and visualized by LC-MS. Signal intensity was assessed as a function of retention time and the peaks corresponding to the three major GPL families are labeled.
